# Solvent‐Desorption‐Induced Formation of a Long‐Period Polymorph Yielding a High‐Performance Porphyrin‐Based Organic Semiconductor

**DOI:** 10.1002/advs.77915

**Published:** 2026-09-27

**Authors:** Kazuya Miyazaki, Keitaro Yamamoto, Mitsuaki Yamauchi, Yoshiyuki Mizuhata, Hiroshi Matsuda, Nobutaka Shioya, Takeshi Hasegawa, Hiroko Yamada

**Affiliations:** ^1^ Institute for Chemical Research Kyoto University Uji Kyoto Japan

**Keywords:** crystal‐structure transformation, molecular packings, organic semiconductors, polymorphism, porphyrinoids

## Abstract

Polymorphism is common in organic semiconductors, yet experimentally accessible crystal structures are often limited to thermodynamically favored phases formed under equilibrium crystallization conditions. Here, we report a structural transformation in single crystals of 5,15‐bisphenyl‐tetrabenzoporphyrin (**Ph‐BP**) triggered by the removal of intercalated *o*‐dichlorobenzene. This transformation induces a 90° rotation of the π‐stacking direction between adjacent layers, giving rise to a previously unobserved long‐period polymorph characterized by the periodic alternation of two distinct herringbone packing motifs. The solvent‐desorption‐induced transformed phase can also be formed on Si/SiO_2_ substrates and affords single‐crystal organic field‐effect transistors exhibiting hole mobilities higher than those of devices based on the previously reported **Ph‐BP** polymorph. These findings suggest that solvent‐desorption‐induced structural transformations provide a viable strategy for accessing hidden polymorphs with enhanced semiconductor performance.

## Graphical abstract

Solvent‐desorption‐induced structural transformation of 5,15‐bisphenyl‐tetrabenzoporphyrin generates a long‐period polymorph, in which the π‐stacking direction rotates by 90° between adjacent layers within the crystal while largely retaining crystallographic integrity. Interlayer CH–π interactions between phenyl substituents stabilize this rotated motif, unlocking a hidden packing phase that yields high‐performance OFETs with an average hole mobility of 3.5 ± 1.2 cm^2^ V^−^^1^ s^−^^1^.

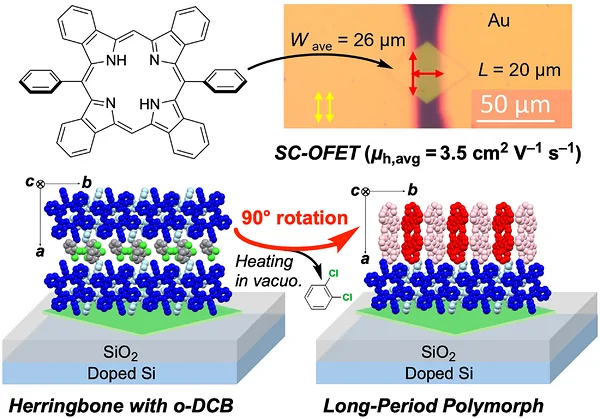

## 1 Introduction

In organic semiconductors (OSCs), charge transport performance is critically governed by molecular packing [1, 2, 3]. Representative packing motifs in OSCs include one‐dimensional (1D) slipped stacking, two‐dimensional (2D) herringbone stacking, and brickwork arrangements [4, 5, 6]. These packing structures are formed through the interplay between molecular shape and intermolecular interactions associated with π–stacked and CH–π contact geometries. Among them, 2D packing is particularly advantageous for organic field‐effect transistor (OFET) applications since charge carriers can conduct through multiple lateral pathways, thereby minimizing the detrimental effect of structural disorder on charge transport [7, 8]. Meanwhile, polymorphism is a common phenomenon in organic compounds since the inherently weak van der Waals intermolecular interactions lead to small energy differences among different polymorphs [9, 10, 11]. Representative examples include high‐performance OSCs such as pentacene [12, 13], thienoacene [14, 15], and phthalocyanine [16, 17, 18], which exhibit polymorphism depending on the crystallization or film fabrication conditions. Thermodynamic stability generally favors lower‐energy polymorphs, so that higher‐energy crystal forms are less frequently obtained under equilibrium crystallization conditions [19], despite their potential for superior charge transport characteristics.

Structural transformations in molecular crystals triggered by external stimuli are intriguing because they can provide access to structures that are otherwise inaccessible by conventional synthetic or crystallization methods [20, 21, 22]. Such transformations can be induced by external stimuli, including light irradiation, heating, and guest uptake or release. In some cases, these processes proceed as single‐crystal‐to‐single‐crystal (SCSC) transformations, in which chemical reactions or structural rearrangements occur while largely preserving the integrity of the single crystal [23, 24]. In such systems, relatively small structural changes are generally favored because large molecular rearrangements often lead to crystal degradation [21]. Metal–organic frameworks (MOFs) and coordination polymers are also representative systems capable of substantial structural reorganization while retaining crystallinity, owing to their robust coordination networks [25, 26, 27]. To date, however, structural transformations involving a collective reorientation of the π–stacking direction within individual molecular layers have rarely been reported in OSCs.

**FIGURE 1:**
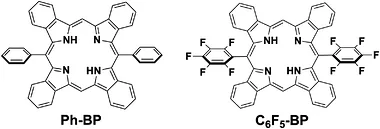
Molecular structures investigated in this study.

Herein, we report a solvent‐desorption‐induced structural transformation in the crystalline state of a rigid, π–extended porphyrin derivative, 5,15‐bisphenyl‐tetrabenzoporphyrin (**Ph‐BP**; Figure 1). This process induced a structural rearrangement into a long‐period polymorph, characterized by adjacent layers whose π–stacking directions are rotated by 90° relative to one another. This transformation was also realized in single crystals grown on Si/SiO_2_ substrates, enabling the fabrication of SC‐OFETs that exhibited a high average hole mobility for porphyrin‐based semiconductors (3.5 ± 1.2 cm^2^ V^−1^ s^−1^). These results demonstrate the potential of solvent‐desorption‐induced crystalline transformations as an approach to accessing polymorphs with favorable charge‐transport properties.

## 2 Results and Discussion

**FIGURE 2:**
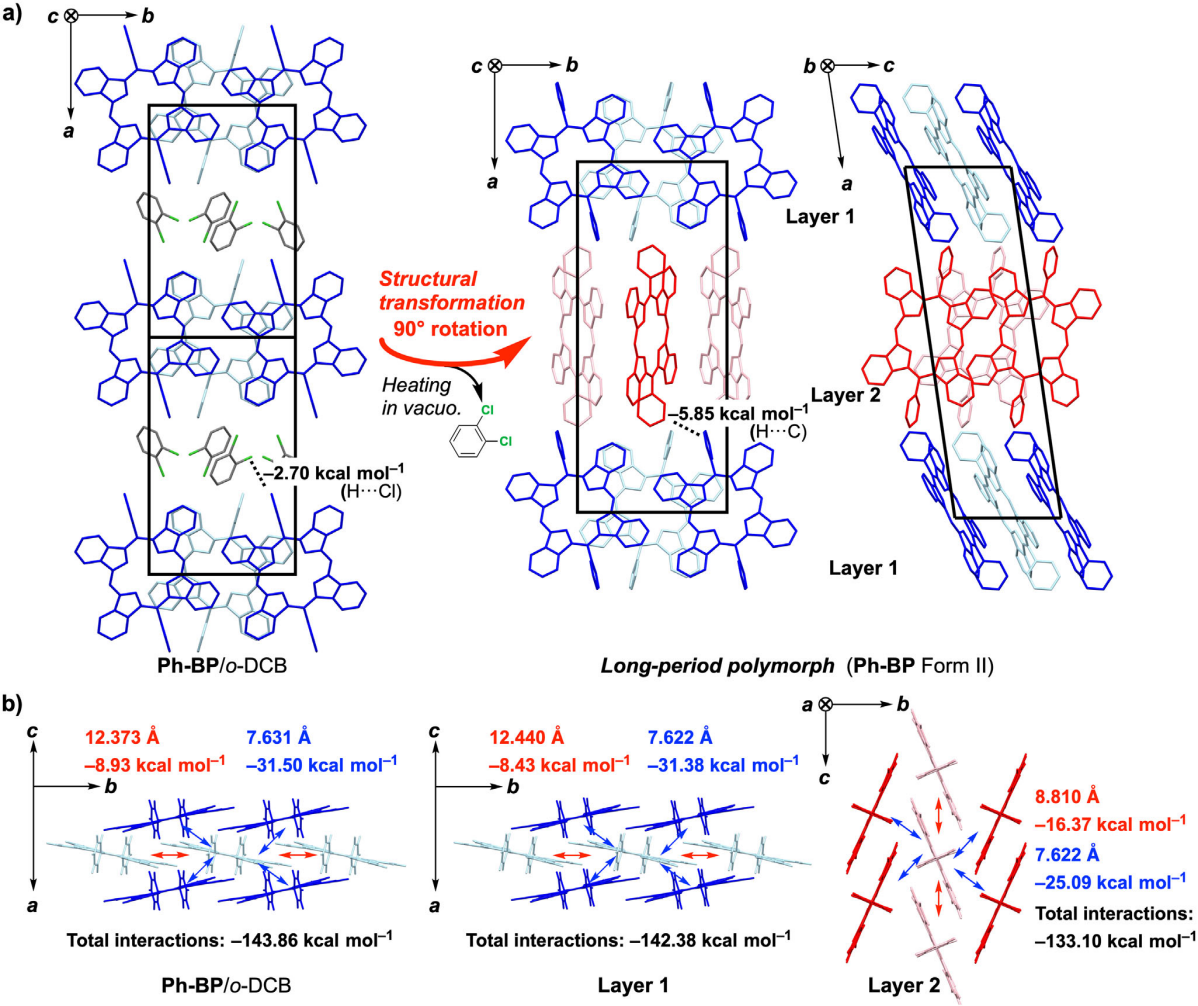
(a) Schematic illustration of the structural transformation from **Ph‐BP**/*o*‐DCB (CCDC: 2401103) to **Ph‐BP** Form II (CCDC: 2512996). Hydrogen atoms are omitted for clarity. (b) Centroid‐to‐centroid distances and intermolecular interaction energies in the packing of **Ph‐BP**/*o*‐DCB (left), Layer 1 (middle), and Layer 2 (right) of **Ph‐BP** Form II. The red and blue double‐headed arrows denote slip‐parallel and T‐shaped pairs, respectively.

**Ph‐BP** was synthesized following the procedure already reported [28]. **Ph‐BP** crystals were obtained by vapor diffusion using *o*‐DCB/THF (1:1 v/v) as a good solvent and *n*‐octane as a poor solvent. X‐ray analysis revealed a solvated lattice (**Ph‐BP**/*o*‐DCB; Figure S1), where *o*‐DCB molecules are intercalated between the herringbone layers (Figure 2a). Refinement of the *o*‐DCB occupancy converged to approximately 0.96, indicating that the solvent sites were nearly fully occupied in the as‐grown crystal. Heating these crystals at 180°C under vacuum (6.7 × 10^−2^ Pa) induced solvent desorption, accompanied by a structural transformation. Notably, while the herringbone motif was preserved, the π–stacking direction rotated by 90° between adjacent layers, forming a long‐period polymorph (**Ph‐BP** Form II). This architecture features a two‐layer crystallographic periodicity arising from distinct herringbone arrangements in adjacent layers. The resultant crystals exhibited minor polycrystallinity, leading to reduced bond‐length precision and relatively higher *R* values in the crystallographic refinement (Table S1). Nevertheless, the refined atomic displacement parameters obtained without applying any restraints, together with the satisfactorily resolved residual peak and hole values, indicate that both the lattice determination and the structural solution remain reliable. In addition, polarized optical microscopy (POM) images recorded before and after vacuum heating at 180°C showed largely uniform birefringence across each crystal, with no apparent deterioration in its optical uniformity (Figure S2). These observations suggest that the crystal retains a substantial degree of crystallographic integrity during the solvent‐desorption‐induced structural transformation, despite a partial loss of crystallinity.

The solvated structure of **Ph‐BP**/*o*‐DCB is stabilized by weak H···Cl contacts between the phenyl substituents and *o*‐DCB molecules (−2.70 kcal mol^−1^), as confirmed by symmetry‐adapted perturbation theory (SAPT) calculations (Figure S3) [29]. **Ph‐BP** Form II also exhibited a herringbone packing arrangement in the *bc* plane and formed a lamellar structure along the *a*‐axis (Figure 2a). In Figure 2, the red and pink molecules, as well as the blue and light‐blue molecules, are related by the glide‐plane symmetry operation of the *P*2_1_/*c* space group, as illustrated in Figure S4. Interestingly, Layer 1 forms a π–stacked arrangement along the *c*‐axis, whereas Layer 2 forms π–stacking along the *b*‐axis, resulting in orthogonal π–stacking directions between adjacent layers. While the herringbone packing structures in Layer 1 remain structurally similar to those observed in **Ph‐BP**/*o*‐DCB, Layer 2 exhibits significantly reduced centroid–centroid distances in the slip‐parallel pair (shown as the red double‐headed arrow), leading to an enhanced 2D packing arrangement (Figure 2b). To further clarify the geometrical differences among these packing motifs, the dihedral angles between the molecular planes and the angles between the molecular long axes for the T‐shaped and slip‐parallel contacts are summarized in Table S2 for Layers 1 and 2 of **Ph‐BP** Form II. These parameters reveal distinct relative orientations of neighboring molecules in the respective packing structures.

The total interaction energies with the six neighboring molecules are −143.86, −142.38, and −133.10 kcal mol^−1^ for **Ph‐BP**/*o*‐DCB, Layer 1, and Layer 2, respectively. Note that these interaction energies are substantially greater in magnitude than that of pentacene (−80.72 kcal mol^−1^), a representative OSC with a 2D herringbone packing (Figure S5). The detailed energy decomposition provided in Table S3 shows that the stabilization energies originate predominantly from dispersion and electrostatic components. These results indicate that the extended π–system and rigid molecular framework of **Ph‐BP** lead to strong intermolecular stabilization associated with π–stacked arrangements and CH–π contacts, which likely help the crystals retain substantial crystallographic integrity during the structural rearrangement. In **Ph‐BP** Form II, intermolecular contacts between the phenyl substituents were also observed across adjacent layers. Specifically, the meta‐hydrogen atom on the phenyl substituent of Layer 2 interacts with the phenyl ring of Layer 1 in an orthogonal geometry, exhibiting a stabilization energy of −5.85 kcal mol^−1^. This value is larger in magnitude than the **Ph‐BP**/*o*‐DCB interaction, suggesting that the formation of this interlayer CH–π interaction contributes to the stabilization of the solvent‐desorbed structure and plays an important role in maintaining the 90° rotated packing arrangement induced by the structural transformation.

**FIGURE 3:**
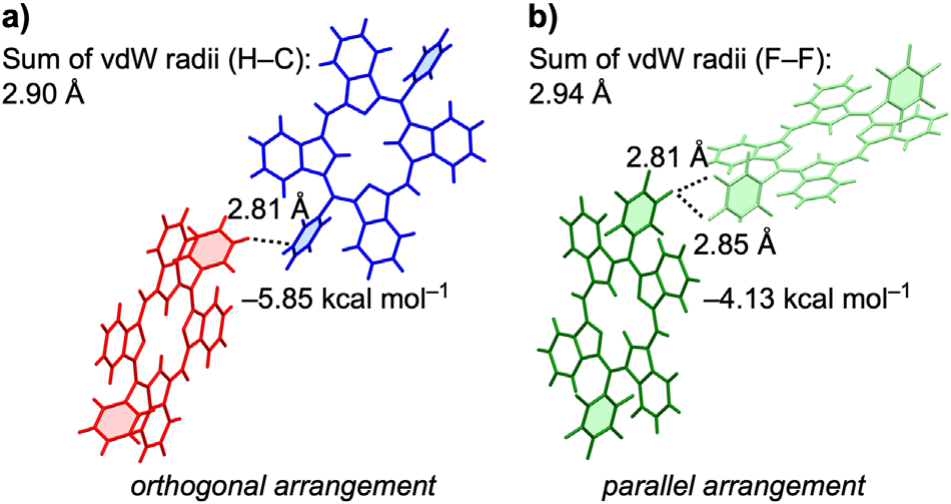
Interlayer intermolecular interactions in (a) **Ph‐BP** Form II and (b) **C_6_F_5_‐BP** bilayer.

To examine the influence of substituents on the resulting packing structure via the structural transformation, we investigated the behavior of **C_6_F_5_‐BP** (molecular structure is shown in Figure 1), in which the phenyl substituents were replaced with pentafluorophenyl groups. Although the pentafluorophenyl group is similar in size to the phenyl group, it possesses an opposite electrostatic potential due to the electron‐withdrawing nature of the fluorine atoms (Figure S6) [30]. Recrystallization of **C_6_F_5_‐BP** under identical conditions to **Ph‐BP** afforded solvated single crystals (**C_6_F_5_‐BP**/*o*‐DCB) isostructural to **Ph‐BP**/*o*‐DCB (Figure S8a). As in the case of **Ph‐BP**, the herringbone packing of **C_6_F_5_‐BP** is mainly stabilized by both dispersion and electrostatic contributions associated with π–stacked arrangements and CH–π contacts (Table S3). The intercalated *o*‐DCB molecules occupy positions nearly identical to those in **Ph‐BP**/*o*‐DCB, stabilized by specific H···F contacts (2.60 Å) with the pentafluorophenyl substituents (Figure S7) [31]. Heating **C_6_F_5_‐BP**/*o*‐DCB under vacuum also led to the release of *o*‐DCB and induced a structural transformation (Figure S8a). In contrast to **Ph‐BP**, the solvent‐desorbed crystal of **C_6_F_5_‐BP** adopts a bilayer herringbone structure (**C_6_F_5_‐BP** bilayer), in which both layers stack along the same axis. The major interlayer interactions in **Ph‐BP** Form II and **C_6_F_5_‐BP** bilayer are extracted and shown in Figure 3, while detailed intermolecular contacts are visualized by Hirshfeld surface analysis in Figure S9 [32]. The phenyl groups in **Ph‐BP** exhibit an “edge‐to‐face” CH–π geometry, which promotes the orthogonal orientation of the π–stacking directions. Conversely, the pentafluorophenyl groups in **C_6_F_5_‐BP** adopt an “edge‐to‐edge” geometry involving weak F···F contacts [33], resulting in a parallel bilayer structure. These observations indicate that substituent interactions contribute to the interlayer arrangements during the structural transformation.

Thermogravimetric analysis (TG‐DTA) revealed endothermic weight losses of 21.2% (48°C–136°C) for **Ph‐BP**/*o*‐DCB and 20.4% (59°C–215°C) for **C_6_F_5_‐BP**/*o*‐DCB, respectively (Figure S10). These values are lower than the theoretical solvent contents (30.7% and 25.9%). Before the TG‐DTA measurement, the crystals were isolated from the recrystallization solvent and subjected to vacuum for 10 min to remove residual surface‐adsorbed solvent. This process likely induced partial desorption of the lattice solvent before the measurement. Indeed, ^1^H NMR analysis of crystals after the same pretreatment revealed that the remaining *o*‐DCB content was approximately 83% of the theoretical value expected for the stoichiometric solvate. The onset of *o*‐DCB desorption below its boiling point (180°C) likely reflects the different molecular environments and intermolecular interactions of *o*‐DCB in the crystal lattice compared with those in the neat liquid. In addition, stabilization associated with the formation of **Ph‐BP** Form II may provide an additional driving force for solvent desorption [34].

**FIGURE 4:**
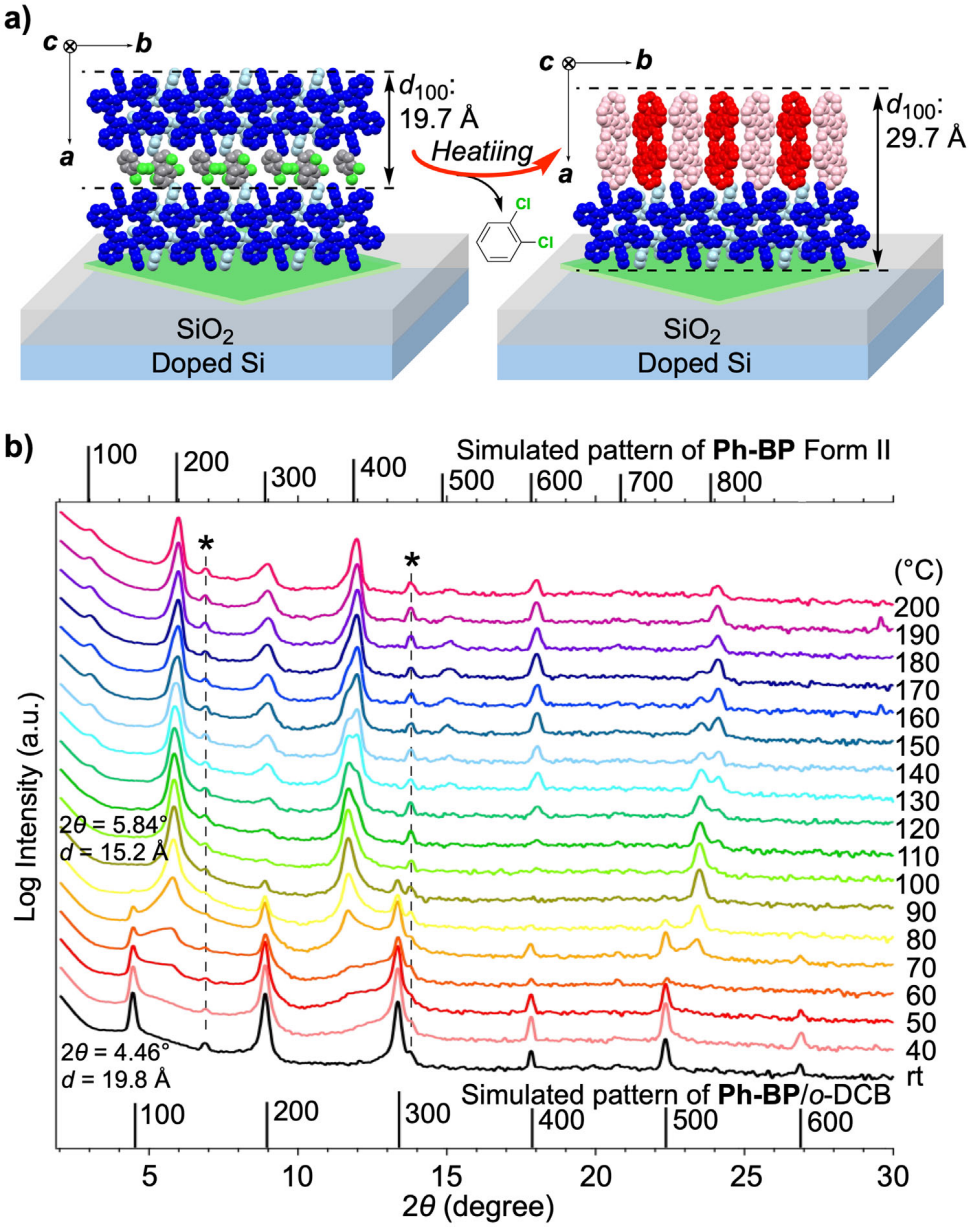
(a) Schematic illustration of the structural transformation from **Ph‐BP**/*o*‐DCB to **Ph‐BP** Form II on the substrate. (b) Out‐of‐plane XRD patterns of **Ph‐BP**/*o*‐DCB single crystals grown on Si/SiO_2_ substrates during stepwise annealing from 40°C to 200°C. Peaks marked with asterisks are assigned to the 100 and 200 reflections of **Ph‐BP** Form I [28] coexisting on the substrate. Their unchanged intensities indicate that this phase was not generated during the solvent‐desorption‐induced transformation.

To further investigate the structural transformation behavior of **Ph‐BP**/*o*‐DCB, out‐of‐plane XRD measurements were performed on an ensemble of single crystals grown on Si/SiO_2_ substrates (**Figure** **4**). Step‐annealing was performed from 40 to 200°C (°C steps, 10 min each), followed by cooling to room temperature for each XRD measurement. The initial XRD pattern exhibited peaks corresponding to the (h00) reflections (h = 1–6) of the bulk single crystal of **Ph‐BP**/*o*‐DCB, indicating that the crystals grown on the Si/SiO_2_ substrate possess the same structure as the bulk single crystal of **Ph‐BP**/*o*‐DCB and are oriented with the a*‐axis perpendicular to the surface. Upon annealing between 50 and 100°C, the original peaks associated with **Ph‐BP**/*o*‐DCB (*d* = 19.8 Å) disappeared, while new peaks corresponding to the *d*‐spacing of 15.2 Å emerged. This reduction in *d*‐spacing reflects a more condensed packing, where the interlayer voids created by *o*‐DCB desorption are subsequently filled. This intermediate phase then underwent subtle lattice adjustments up to 200°C, where the diffraction peaks shifted slightly toward higher angles and the emergence of distinct odd (h00) reflections confirmed improved agreement with the simulated **Ph‐BP** Form II (Figure S11). Note that no significant endothermic or exothermic signals were observed in the DTA curve after the endothermic desorption of *o*‐DCB (Figure S10). This absence of thermal transitions suggests that no major structural reorganization occurs during this stage, supporting the view that the transition from the intermediate phase to **Ph‐BP** Form II is a subtle optimization of the lattice parameters rather than a discrete phase transformation. To compare the relative energies of the **Ph‐BP** polymorphs, we evaluated their lattice energies using periodic density functional theory (DFT) calculations [19]. The results revealed that **Ph‐BP** Form II is 0.88 kcal mol^−1^ higher in energy than the previously reported **Ph‐BP** polymorph (**Ph‐BP** Form I) obtained from THF and *n*‐octane (Figure S12) [28], suggesting that this higher‐energy phase can be accessed through the nonequilibrium process of solvent desorption.

Temperature‐dependent out‐of‐plane XRD measurements were also performed for **C_6_F_5_‐BP** crystals by stepwise annealing in 20°C increments for 10 min at each temperature (Figure S13). The diffraction pattern measured at room temperature did not agree with the simulated pattern of **C_6_F_5_‐BP**/*o*‐DCB. Although the cause of this discrepancy is unclear, structural changes associated with solvent loss may be one possible explanation. At approximately 100°C, a series of (00l) reflections (l = 2, 4, 6, 8, and 10) appeared at positions close to those simulated for the bilayer structure of **C_6_F_5_‐BP**. Upon further heating, new peaks began to appear slightly on the lower‐angle side at 160°C. As the temperature increased to 200°C, these peaks gradually intensified, while the peaks observed at 100°C decreased in intensity, indicating a slight increase in the corresponding periodic spacing.

**FIGURE 5:**
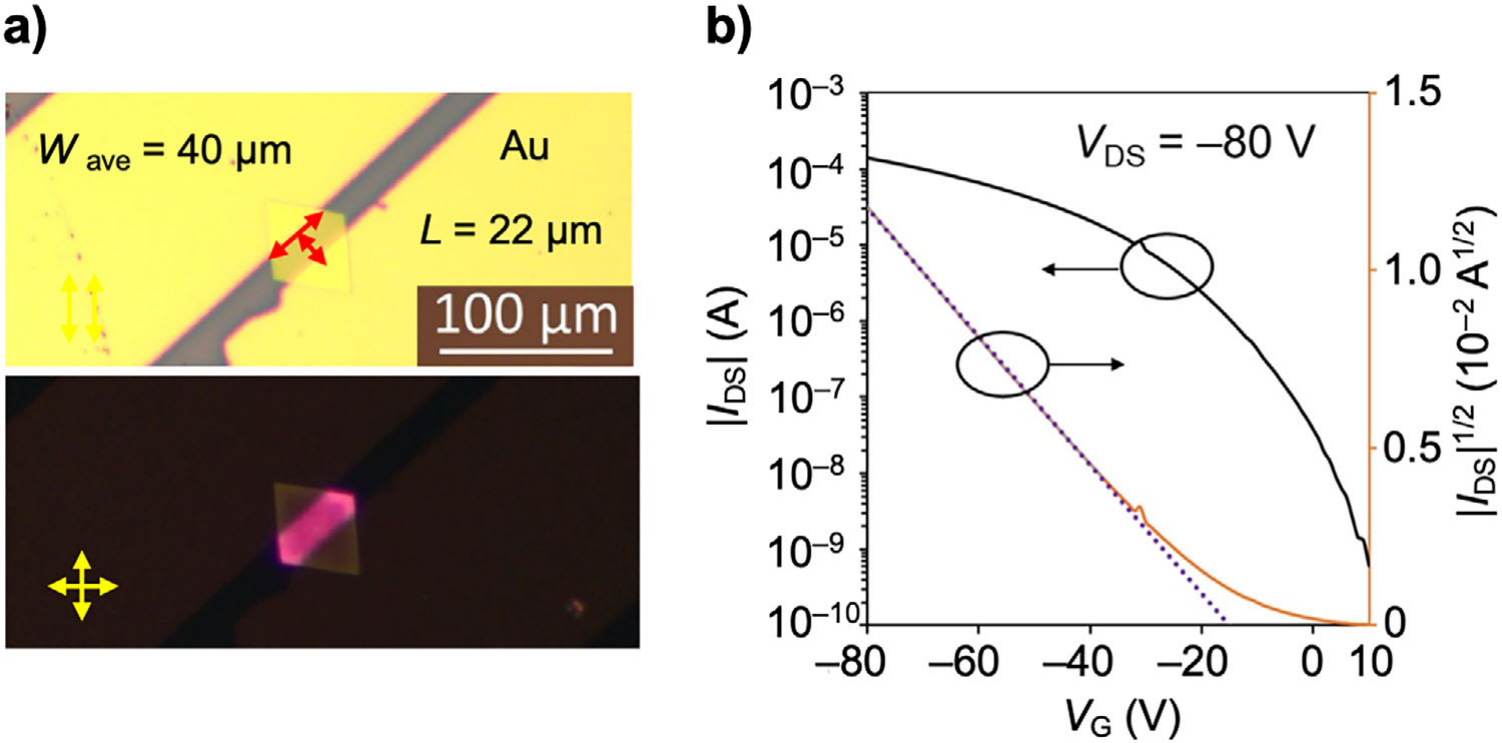
(a) Optical micrograph (top) and polarized optical micrograph (bottom) and (b) transfer characteristics of a representative SC‐OFET based on **Ph‐BP**.

To evaluate the semiconducting properties of **Ph‐BP**, single‐crystal organic field‐effect transistors (SC‐OFETs) were fabricated in a bottom‐gate top‐contact (BGTC) configuration. Following the thermal evaporation of Au source and drain electrodes in vacuo onto **Ph‐BP**/*o*‐DCB crystals, the XRD pattern revealed that the heat generated during the deposition had already induced the structural transformation. This profile is consistent with the out‐of‐plane XRD pattern of the intermediate phase observed at 100°C in the annealing experiments described above, rather than that of the fully transformed **Ph‐BP** Form II (Figure S14). As shown in Figure 5a, the electrodes were patterned with a channel length of 20 µm along the long diagonal of the rhombic crystal, corresponding to carrier transport along the *b*‐axis. The crystal thicknesses of eight device samples were measured by laser scanning microscopy, giving a mean thickness of 101.5 ± 18.0 nm (mean ± SD, n = 8; Figure S15). The charge transport characteristics were measured under ambient conditions, and the transfer and output curves of a representative device are shown in Figure 5b and Figure S16. Excluding the exceptional device exhibiting a mobility of 11.3 cm^2^ V^−1^ s^−1^, the remaining 35 devices exhibited an average hole mobility of 3.5 ± 1.2 cm^2^ V^−1^ s^−1^ in the saturation regime. The individual device parameters and the device‐to‐device distribution of the field‐effect mobility, including one exceptionally high value of 11.3 cm^2^ V^−1^ s^−1^, are provided in Table S4 and Figure S17, respectively. The large variation in the on/off ratio primarily stems from differences in the off‐state current. In one device with a relatively high off‐state current, evacuation substantially reduced this current (Figure S18), suggesting a contribution from ambient p‐doping by adsorbed oxygen or moisture. POM images of representative fabricated devices revealed spatial variations in optical uniformity, suggesting that local differences in crystal quality may contribute to the device‐to‐device mobility variations (Figure S19). To assess the validity of the extracted hole mobilities, the Y‐function method was applied to 10 devices using transfer characteristics measured in the linear regime at *V*_DS_ = −5 V (Figure S20) [35, 36]. The mobilities obtained by the Y‐function method were somewhat lower than those determined by conventional saturation‐regime analysis, but remained within a comparable range (Table S4), supporting the overall validity of the mobility values obtained from the conventional analysis. Notably, the average mobility obtained from the conventional saturation‐regime analysis (3.5 ± 1.2 cm^2^ V^−1^ s^−1^) is markedly higher than that previously reported for **Ph‐BP** Form I (0.73 ± 0.28 cm^2^ V^−1^ s^−1^; maximum: 1.21 cm^2^ V^−1^ s^−1^) [28]. To the best of our knowledge, the hole mobilities obtained in this study also exceed those reported for representative tetrabenzoporphyrin‐based SC‐OFETs [37]. In contrast, **C_6_F_5_‐BP** crystals did not exhibit clear field‐effect characteristics when conventional Au source and drain electrodes were employed. This was possibly attributed to the electron‐withdrawing pentafluorophenyl groups, which may hinder efficient hole injection required for p‐type OFET operation. To improve hole injection, a 5‐nm‐thick MoO_3_ interlayer was introduced between the Au electrodes and the **C_6_F_5_‐BP** crystal. The resulting devices exhibited p‐type transistor characteristics, with hole mobilities on the order of 10^−2^ cm^2^ V^−1^ s^−1^ (Figure S21 and Table S5).

Meanwhile, devices configured for transport along the *c*‐axis exhibited a maximum mobility of 2.0 cm^2^ V^−1^ s^−1^ (average of 4 devices: 1.5 ± 0.54 cm^2^ V^−1^ s^−1^) (Figure S22 and Table S6). As a theoretical reference, transfer integrals were calculated based on the **Ph‐BP** Form II structure, revealing substantial intralayer electronic couplings between multiple molecular pairs in both Layer 1 and Layer 2 (Figure S23). The differences in the transfer integrals between the two layers arise from their distinct molecular packing arrangements. In Layer 1, both coupling pathways have vector components along the *b*‐axis, whereas Layer 2 contains one major pathway with a component along the *b*‐axis and another oriented along the *c*‐axis. Therefore, both layers can potentially contribute to charge transport along the *b*‐axis, while the newly formed Layer 2 additionally provides a *c*‐axis‐directed pathway. In contrast, the interlayer transfer integrals were below 1 meV and were substantially smaller than the intralayer values (Figure S24), suggesting that charge transport is dominated by intralayer pathways.

There have been a few reports of 2D packing arrangements where the packing directions are mutually orthogonal between layers [38, 39]. **Ph‐BP** Form II exhibits a 2D herringbone packing in which the π–stacking directions of adjacent layers are oriented orthogonally to each other, a configuration that may facilitate charge transport along multiple directions within the crystal. To experimentally evaluate the anisotropy of charge carrier mobility in the long‐period polymorph structure, gold source and drain electrodes were thermally evaporated through a cross‐shaped shadow mask, enabling measurements along the *b*‐axis, *c*‐axis (Figure S25a), and the direction rotated by 45° from the *b*‐axis (Figure S25b). As this electrode configuration differs from that used for the mobility measurements discussed above, the absolute mobility values are not directly comparable. However, the devices exhibited mobilities of 2.7 ± 0.1 cm^2^ V^−^^1^ s^−^^1^ along the *b*‐axis, 1.4 ± 0.6 cm^2^ V^−^^1^ s^−^^1^ along the *c*‐axis, and 2.1 ± 0.5 cm^2^ V^−^^1^ s^−^^1^ along the 45° direction (Table S7). These results suggest that the long‐period polymorph supports charge transport along multiple crystallographic directions and are consistent with contributions from the orthogonally oriented π–stacking.

## 3 Conclusion

In conclusion, we demonstrated solvent‐desorption‐induced structural transformations in BP‐based crystals. Upon solvent release, **Ph‐BP** was converted into a long‐period polymorph in which the π–stacking directions of adjacent layers are rotated by 90°. This transformation was also realized in single crystals grown on Si/SiO_2_ substrates. The SC‐OFETs exhibited a high average hole mobility of 3.5 ± 1.2 cm^2^ V^−1^ s^−1^. The mobility‐anisotropy measurements support the presence of multiple transport pathways within the crystal. These findings suggest the potential of solvent‐desorption‐induced structural transformations as an approach to accessing hidden packing phases in OSCs.

## 4 Experimental Section

### 4.1 Materials Synthesis

The synthetic procedure of **C_6_F_5_‐BP** is detailed in the Supporting Information (Scheme S1 and Figures S26–S33).

### 4.2 X‐Ray Diffraction Studies

Single crystals of **Ph‐BP**/*o*‐DCB were obtained by vapor diffusion using *o*‐DCB/THF (1:1 v/v) as a good solvent and *n*‐octane as a poor solvent. Heating these crystals under vacuum (6.7 × 10^−2 ^Pa) at 180°C for 60 min afforded the solvent‐desorbed single crystals, **Ph‐BP** Form II. Similarly, single crystals of **C_6_F_5_‐BP**/*o*‐DCB were grown by vapor diffusion using *o*‐DCB/THF (1:1 v/v) as a good solvent and *n*‐octane as a poor solvent. Upon heating under vacuum (6.7 × 10^−2^ Pa) at 180°C for 60 min, these crystals were converted into the corresponding solvent‐desorbed single crystals, **C_6_F_5_‐BP** bilayer. The intensity data were collected at 90 K on a Bruker D8 VENTURE system (PHOTONIII 14 with IµS Diamond) using Mo Kα radiation (*λ* = 0.71073 Å). The intensity data were corrected for Lorentz and polarization effects and for absorption (multi‐scan). The structures were solved by SHELXT‐2018/2 [40] and refined by least‐squares calculations on *F* ^2^ for all reflections (SHELXL‐2025/1) [41]. All non‐hydrogen atoms were refined anisotropically. All calculations were performed by using Yadokari‐XG [42] and Olex2 1.5 [43]. The crystallographic data are summarized in Table S1.

In the crystals obtained after thermal conversion, slight polycrystallinity was detected. In addition, the loss of dichlorobenzene, a heavy‐atom solvent, resulted in weakened diffraction intensities and, as a consequence of the polycrystalline nature, distorted diffraction spots accompanied by extra reflections. Accordingly, the checkCIF reports list the following alerts for both sets of crystals:

- **PLAT340_ALERT_3_B** Low Bond Precision on C─C Bonds …………… 0.01431 Å (**Ph‐BP** Form II) / 0.01352 Å (**C_6_F_5_‐BP** bilayer)

For the **Ph‐BP** Form II crystals,

- **PLAT084_ALERT_3_B** High wR2 Value (i.e. > 0.25) ………………. 0.36

was also noted.

Even so, the refined displacement parameters—obtained without applying any restraints (except for an EADP constraint at one site in the **C_6_F_5_‐BP** bilayer crystals)—together with the satisfactorily resolved residual peak/hole values indicate that both the lattice determination and the structural solution are reliable.

### 4.3 Out‐of‐Plane XRD Measurements

Out‐of‐plane XRD measurements of single crystals on Si/SiO_2_ substrates were performed using a Rigaku SmartLab X‐ray diffractometer with a Cu Kα source (*λ* = 1.5418 Å) in the *θ*/2*θ* scan mode with a speed of 1° min^−1^ and a step interval of 0.02°.

### 4.4 TG‐DTA Measurements

TG‐DTA measurements were carried out on a Shimadzu DTG‐60 instrument at a heating rate of 10°C min^−^^1^ under an Ar atmosphere.

### 4.5 Fabrication and Evaluation of SC‐OFET

All SC‐OFET devices were fabricated in a top‐contact bottom‐gate configuration on a heavily n‐doped Si wafer with a 300 nm‐thick thermally grown SiO_2_ layer (*C*_i_ = 11.5 nF cm^−2^). The Si/SiO_2_ substrates were cleaned with deionized water, acetone, and 2‐propanol for 10 min each in an ultrasonic bath. Substrates were dried with a flow of N_2_ gas and then treated by UV–O_3_ cleaner (Filgen UV253V8) for 45 min. Single crystals for SC‐OFET measurements were prepared as follows: The **Ph‐BP** solution (0.25 mg mL^−^^1^) in *o*‐DCB/THF (1:1, v/v) was drop‐cast onto a UV–O_3_‐treated Si/SiO_2_ substrate and subjected to *n*‐octane vapor diffusion as a poor solvent for 3 days at 22°C, affording single crystals of **Ph‐BP**/*o*‐DCB. The shadow mask was subsequently placed on top of the single crystals and fixed in position using polyimide tape. Then, Au source and drain electrodes (30 nm) were vacuum‐deposited at 0.1 Å s^−1^ under the pressure of 5 × 10^−4^ Pa through a shadow mask on the single crystal. For **C_6_F_5_‐BP** devices, a 5‐nm‐thick MoO_3_ interlayer was vacuum‐deposited at 0.1 Å s^−1^ onto the single crystals prior to Au deposition. The heat generated during the deposition process led to the desorption of *o*‐DCB from the **Ph‐BP** crystal, resulting in its transformation into the solvent‐desorbed **Ph‐BP** phase. The thickness of the single crystals was measured using a nanosurface profilometer equipped with a confocal laser microscope (Shimadzu, SFT‐4500). The source–drain channel length (*L*) and width (*W*) of each device were measured independently using Zeiss Axio Scope.A1 optical microscope. The output and transfer characteristics of the OFETs were measured using a Thermal Block SB‐MCPS‐NAT prober system and a Keithley 2400 source measure unit at room temperature in air.

The field‐effect hole mobilities (*µ*_h_) of the OFETs were determined from the forward transfer curve in the saturation regime (*V*_DS_ = −80 V) using the following equation:

(1) $$\left|I_{\mathrm{DS}}\right|=\left(\mu WC_{i}/2L\right)\left(V_{G}\;-\;V_{\mathrm{th}}\right)$$

where *I*_DS_ is the drain–source current and *V*_DS_, *V*_G_, and *V*_th_ are the drain–source voltage, gate voltage, and threshold voltage, respectively. The mobility was extracted from the slope of the linear region of each |*I*_DS_|^1/2^–*V*_G_ plot. The on/off current ratios (*I*_on_/*I*_off_) were determined from the *I*_DS_ at *V*_G_ = 0 V (*I*_off_) and *V*_G_ = –80 V (*I*_on_).

Y‐function analysis was performed using the forward transfer curves measured in the linear regime at *V*_DS_ = −5 V, according to the following equation:

$$Y=|I_{\mathrm{DS},\mathrm{lin}}|/g_{m}^{1/2}={\left[\left(W/L\right)\mu_{h,Y}C_{i}|V_{\mathrm{DS}}|\right]}^{1/2}|V_{G}-V_{T}|$$

where *g*_m_ is the transconductance defined as *g*_m_ = ∂*I*_DS_ /∂*V*_G_, *W* and *L* are the channel width and length, respectively, *C*_i_ is the gate‐dielectric capacitance per unit area, and *µ*_h,Y_ is the mobility extracted by the Y‐function method. The mobility was determined from the slope of the *Y*–*V*_G_ plot by linearly fitting an approximately linear region for each device.

### 4.6 Theoretical Calculations

Intermolecular interaction energies were evaluated using symmetry‐adapted perturbation theory (SAPT) at the jun‐cc‐pVDZ level with the PSI4 program [44]. Dimer geometries were extracted directly from the crystal structures to clarify the contributions of dispersion, electrostatic, and induction interactions stabilizing the packing motifs. Hirshfeld surface analyses were carried out using the CrystalExplorer 21.5 program to visualize intermolecular contacts. The relative energies of the **Ph‐BP** polymorphs were evaluated using periodic density functional theory (DFT) calculations performed with Quantum ESPRESSO [45]. The experimentally determined crystal structures were used as initial geometries, and only the atomic positions were fully optimized while keeping the lattice parameters fixed, without symmetry constraints. The Perdew–Burke–Ernzerhof (PBE) exchange–correlation functional [46] and projector augmented wave (PAW) pseudopotentials [47] were employed. Brillouin‐zone sampling was performed using Monkhorst–Pack *k*‐point meshes of 2 × 3 × 3 for the **Ph‐BP** Form II and 3 × 4 × 2 for the **Ph‐BP** Form I. The *k*‐point meshes were chosen to ensure comparable sampling density in reciprocal space for unit cells of different sizes. The total energies after atomic‐position relaxation were used to compare the lattice stabilities of the different polymorphs. Intermolecular electronic couplings (transfer integrals, *t*) were calculated for molecular dimers extracted from the crystal structures using ADF program with the PBE functional and the TZ2P basis set [48]. Molecular geometry optimizations for ESP plots were carried out using density functional theory at the B3LYP/6‐311+G(d) level with Grimme's D3 dispersion correction with Becke–Johnson damping (GD3BJ), as implemented in Gaussian 16 [49]. Frequency analyses confirmed the absence of imaginary frequencies.

## Author Contributions

**Kazuya Miyazaki**: investigation, methodology, data curation, visualization, writing – original draft, formal analysis, conceptualization. **Keitaro Yamamoto**: conceptualization, methodology, validation, visualization, writing – review and editing, funding acquisition, writing – original draft, formal analysis, data curation. **Mitsuaki Yamauchi**: conceptualization, writing – review and editing, methodology, funding acquisition. **Yoshiyuki Mizuhata**: methodology, investigation, resources, writing – review and editing, formal analysis. **Hiroshi Matsuda**: investigation, writing – review and editing, methodology. **Nobutaka Shioya**: methodology, writing – review and editing, resources. **Takeshi Hasegawa**: methodology, resources, writing – review and editing. **Hiroko Yamada**: conceptualization, supervision, project administration, funding acquisition, resources, writing – review and editing, methodology, validation.

## Funding

This work was supported by the Japan Society for the Promotion of Science (JSPS) KAKENHI (Grant No. JP 24K17738 to K.Y., JP 25K01751 and JP 26K21862 to H.Y., and JP 26K01474 to M.Y.); the Grant‐in‐Aid for Transformative Research Areas “Dynamic Exciton” (JP 20H05833 to H.Y.) and “Photokineticism” (JP 26H00381 to H.Y.); the Iketani Science and Technology Foundation (to H.Y.); and the JST SPRING (Grant No. JPMJSP2110 to K.M.); and JKA through “Promotion Project of Keirin and Auto Race” (Grant No. 2026M‐232).

## Conflicts of Interest

The authors declare no conflicts of interest.

## Supporting information




**Supporting File**: advs77915‐sup‐0001‐SuppMat.pdf.

## Data Availability

The data that support the findings of this study are available from the corresponding author upon reasonable request.
