# Supplementary material for: Solvent‐Desorption‐Induced Formation of a Long‐Period Polymorph Yielding a High‐Performance Porphyrin‐Based Organic Semiconductor
Source: Adv Sci (Weinh). 2026 Sep 27:e77915. Online ahead of print. doi: 10.1002/advs.77915 (PMC13616281; doi:10.1002/advs.77915)
Supplement: Supplementary file 1 [file ADVS-9999-e77915-s001.pdf]

Solvent-Desorption-Induced Formation of a Long-Period Polymorph Yielding a High-Performance Porphyrin-Based Organic Semiconductor

Kazuya Miyazaki, Keitaro Yamamoto, Mitsuaki Yamauchi, Yoshiyuki Mizuhata, Hiroshi Matsuda, Nobutaka Shioya, Takeshi Hasegawa, and Hiroko Yamada**

Table of Contents

1. Synthesis and characterization .... Page 2
2. Single-crystal X-ray crystallographic analysis .... Page 3–4
3. Supplementary figures and tables .... Page 5–18
4. NMR spectra .... Page 19–21
5. HR-MS data .... Page 22
6. Optimized coordinates .... Page 23–26
7. References .... Page 27

1. Synthesis and characterization

All reagents and solvents were purchased from FUJIFILM Wako, Kanto Chemical, Nacalai Tesque, Tokyo Chemical Industry (TCI), or Sigma-Aldrich, and were used as received. Thin-layer chromatography (TLC), silica gel column chromatography, and alumina column chromatography were performed on Art. 105554 silica gel plates (Merck KGaA), silica gel 60N (Kanto Chemical), and Aluminium oxide 90 active neutral (Sigma-Aldrich), respectively. Recycling preparative GPC-HPLC was performed on a Japan Analytical Industry LC-9225NEXT equipped with a series of JAIGEL-1H and 2H (Φ40 mm, $CHCl_3$ eluent, flow rate: 6.0 mL min^{-1}). ^{1}H and ^{13}C NMR spectra were recorded using Bruker Avance NEO 400, Bruker Avance III 600, and Bruker Avance III 800 spectrometers at ambient temperature. Chemical shifts of ^{1}H and ^{13}C NMR signals are quoted to tetramethylsilane (δ = 0.00 ppm) as an internal standard, and those of ^{19}F NMR signals are referenced to $CFCl_3$ (δ = 0.00 ppm) as an external standard. High-resolution mass spectra (HR-MS) were collected using a Bruker solariX FT-ICR mass spectrometer equipped with a MALDI source (matrix: DCTB; internal calibrant: CsI).

Compound **1** was prepared by the reported procedure.[1]

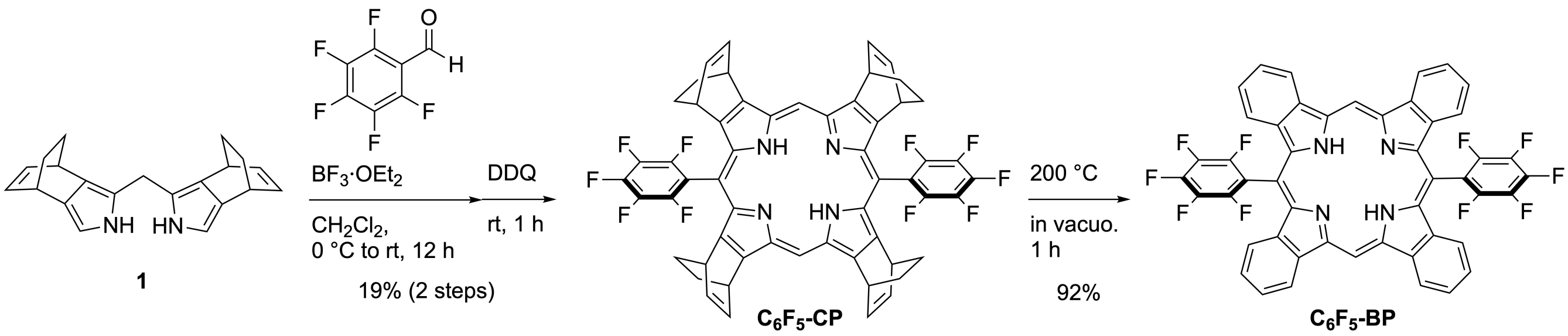

Scheme S1. Synthetic route of **C_6F_5-BP**.

Bicyclo[2.2.2]octadiene-fused 5,15-bis(pentafluorophenyl)-porphyrin (**C_6F_5-CP**):
A solution of compound **1** (441.7 mg, 1.46 mmol) in CH_2Cl_2 (150 mL) was deoxygenated by bubbling argon gas for 30 min. After the addition of pentafluorobenzaldehyde (315.5 mg, 1.60 mmol), boron trifluoride diethyl ether complex ($BF_3{\cdot}OEt_2$, 0.058 mL, 0.58 mmol) was added dropwise to the mixture at 0 °C. The resulting mixture was stirred for 12 h at 0 °C. 2,3-Dichloro-5,6-dicyano-*p*-benzoquinone (DDQ, 678.2 mg, 2.79 mmol) was added to the mixture, and the solution was stirred for 1 h at room temperature. Then, the solvent was removed under reduced pressure. The residue was purified by alumina column chromatography ($CHCl_3$) and silica gel column chromatography (CH_2Cl_2/hexane 1:1, R_f = 0.45) to give **C_6F_5-CP** as a red solid in 19% yield (130.9 mg, 0.14 mmol). Note that this product was isolated as a mixture of stereoisomers regarding the orientation of the bicyclo[2.2.2]octadieno units.
^{1}H NMR ($CDCl_3$, 600 MHz): δ (ppm) = 10.42 (d, J = 2.7 Hz, 2H), 7.08 - 7.06 (m, 4H; Ar H), 6.84 - 6.81 (m, 4H; Ar H), 5.75 (d, J = 5.6 Hz, 4H; Ar H), 4.01 (d, J = 3.3 Hz, 4H; Ar H), 2.15 - 1.68 (m, 16H; CH_2), –3.77 (s, 2H; NH).
^{13}C NMR ($CDCl_3$, 151 MHz): δ (ppm) = 152.1, 147.0, 146.9, 139.7, 137.9, 136.5, 136.4, 136.3, 98.2, 37.8, 36.1, 29.6, 27.3, 27.2.
^{19}F NMR ($CDCl_3$, 565 MHz): δ (ppm) = –136.2 - –137.9 (m), –151.9 (t, J = 20.8 Hz), –162.0 - –162.6 (m).
HR-MS (MALDI) *m/z*: $[M]^+$ calcd for $C_{56}H_{36}F_{10}N_4$ 954.27748, found 954.27702.

5,15-Bis(pentafluorophenyl)-tetrabenzoporphyrin (**C_6F_5-BP**):
C_6F_5-CP (57.3 mg, 60.0 μmol) was heated at 200 °C for 1 h under vacuum in a glass tube oven to give **C_6F_5-BP** as a green solid in 92% yield (46.6 mg, 55.4 μmol). **C_6F_5-BP** was further purified by recrystallization using o-DCB and n-octane to obtain a highly pure material for measurements and fabrication of OFETs.
^{1}H NMR ($CDCl_3$, 600 MHz): δ (ppm) = 11.10 (s, 2H), 9.69 (d, J = 8.0 Hz, 4H; Ar H), 8.20 (t, J = 7.3 Hz, 4H; Ar H), 7.98 (t, J = 7.3 Hz, 4H; Ar H), 7.72 (d, J = 8.0 Hz, 4H; Ar H), –1.17 (s, 2H; NH).
^{13}C NMR ($CDCl_3$, 151 MHz): δ (ppm) = 141.5, 139.8, 139.4, 136.0, 128.6, 127.8, 122.3, 121.8, 94.0.
^{19}F NMR ($CDCl_3$, 565 MHz): δ (ppm) = –136.4 - –136.5 (m), –151.0 (t, J = 20.3 Hz), –160.0 - –160.1 (m).
HR-MS (MALDI) *m/z*: $[M]^+$ calcd for $C_{48}H_{20}F_{10}N_4$ 842.15228, found 842.15178.

2. Single-crystal X-ray crystallographic analysis

Table S1. Crystallographic data for **Ph-BP**/*o*-DCB, **Ph-BP** Form II, **C_6F_5-BP**/*o*-DCB, and **C_6F_5-BP** bilayer.

	**Ph-BP**/*o*-DCB	**Ph-BP** Form II	**C_6F_5-BP**/*o*-DCB	**C_6F_5-BP** bilayer
Empirical formula	$C_{60}H_{38}Cl_4N_4$	$C_{48}H_{30}N_4$	$C_{60}H_{28}Cl_4F_{10}N_4$	$C_{48}H_{20}F_{10}N_4$
Formula weight	956.74	662.76	1136.66	842.68
Temperature/K	90(2)	90(2)	90(2)	90(2)
Crystal system	monoclinic	monoclinic	monoclinic	orthorhombic
Space group	$P2_1/c$	$P2_1/c$	$P2_1/c$	$P2_12_12_1$
a/Å	20.1203(11)	29.966(4)	20.5563(15)	9.8497(13)
b/Å	12.3734(7)	12.5810(18)	12.3375(8)	12.2041(17)
c/Å	8.9353(4)	8.9209(13)	9.4982(7)	30.868(4)
α/°	90	90	90	90
β/°	101.3480(10)	98.115(7)	103.045(2)	90
γ/°	90	90	90	90
Volume/Å^3	2181.0(2)	3329.5(8)	2346.7(3)	3710.6(9)
Z	2	4	2	4
ρ_{calc}g/cm^3	1.457	1.322	1.609	1.508
μ/mm^{-1}	0.321	0.078	0.342	0.125
F(000)	988.0	1384.0	1148.0	1704.0
Crystal size/mm^3	0.18 × 0.12 × 0.09	0.242 × 0.181 × 0.068	0.254 × 0.139 × 0.051	0.23 × 0.14 × 0.05
2θ range for data collection/°	3.886 to 56.628	4.118 to 50.994	4.068 to 56.602	4.254 to 54.994
Index ranges	$-26 \le h \le 26$, $-16 \le k \le 16$, $-11 \le l \le 11$	$-36 \le h \le 36$, $-15 \le k \le 14$, $-8 \le l \le 10$	$-27 \le h \le 27$, $-16 \le k \le 16$, $-12 \le l \le 12$	$-12 \le h \le 12$, $-15 \le k \le 15$, $-40 \le l \le 40$
Reflections collected	70032	26626	75284	77495
Independent reflections	5419 [R_{int} = 0.1066, R_{sigma} = 0.0489]	6199 [R_{int} = 0.0723, R_{sigma} = 0.0973]	5839 [R_{int} = 0.0831, R_{sigma} = 0.0366]	8507 [R_{int} = 0.1765, R_{sigma} = 0.1275]
Data/restraints/parameters	5419/0/383	6199/0/469	5839/0/355	8507/0/565
Goodness-of-fit on F^2	1.025	1.196	1.016	1.105
Final R indexes [I>=2σ (I)]	R_1 = 0.0429, wR_2 = 0.0910	R_1 = 0.1511, wR_2 = 0.3237	R_1 = 0.0365, wR_2 = 0.0813	R_1 = 0.0912, wR_2 = 0.1717
Final R indexes [all data]	R_1 = 0.0767, wR_2 = 0.1071	R_1 = 0.2109, wR_2 = 0.3633	R_1 = 0.0572, wR_2 = 0.0910	R_1 = 0.1783, wR_2 = 0.2141
Largest diff. peak/hole / e Å^{-3}	0.32/-0.35	0.49/-0.40	0.29/-0.38	0.37/-0.42
Flack parameter	–	–	–	0.0(5)
CCDC number	2401103	2512996	2512998	2512997

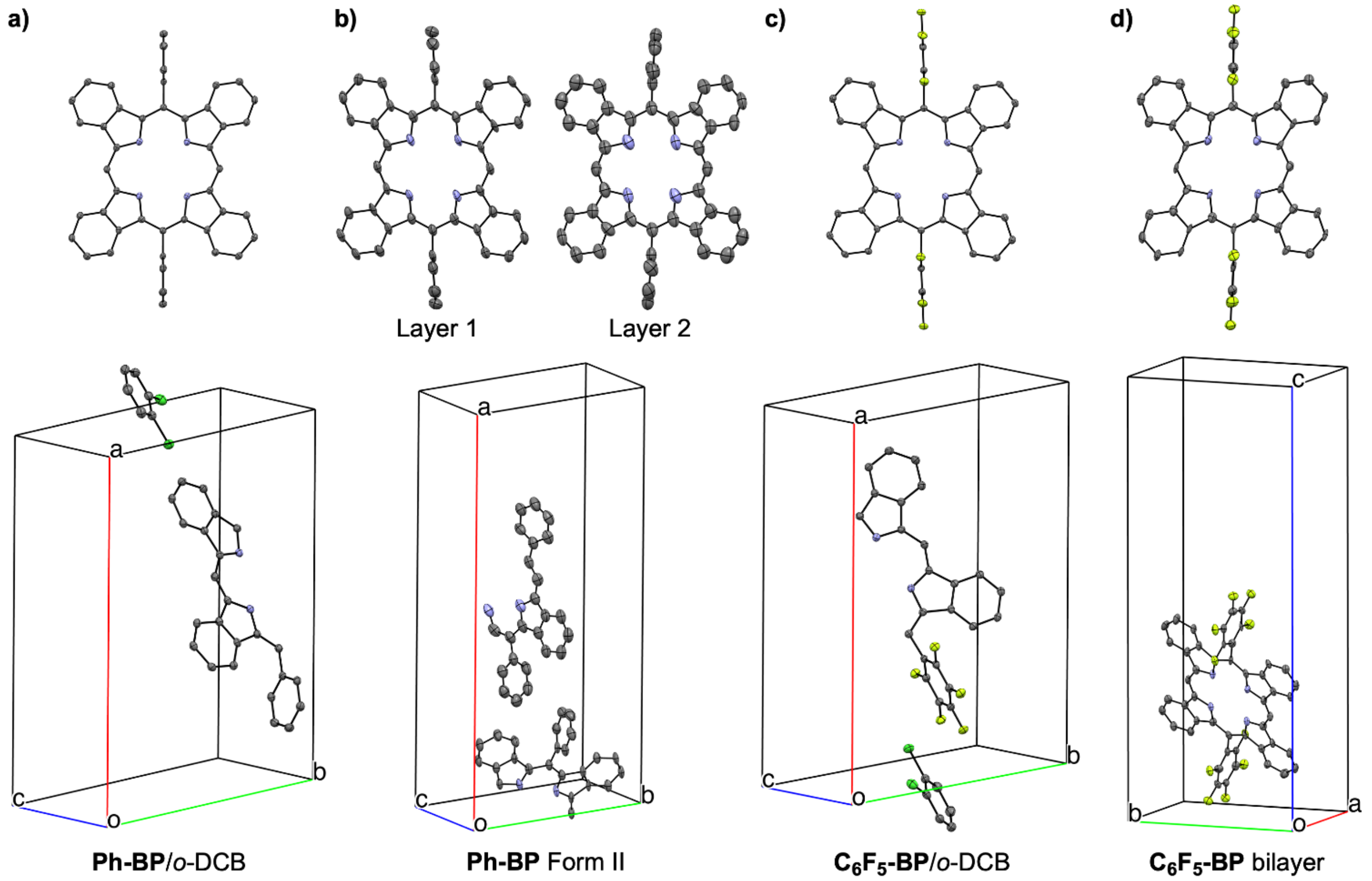

Figure S1. Top views of the molecular structures and asymmetric units of a) **Ph-BP**/*o*-DCB, b) **Ph-BP** Form II, c) **C_6F_5-BP**/*o*-DCB, and d) **C_6F_5-BP** bilayer. Thermal ellipsoids are drawn at the 50% probability level. Hydrogen atoms are omitted for clarity.

3. Supplementary figures and tables

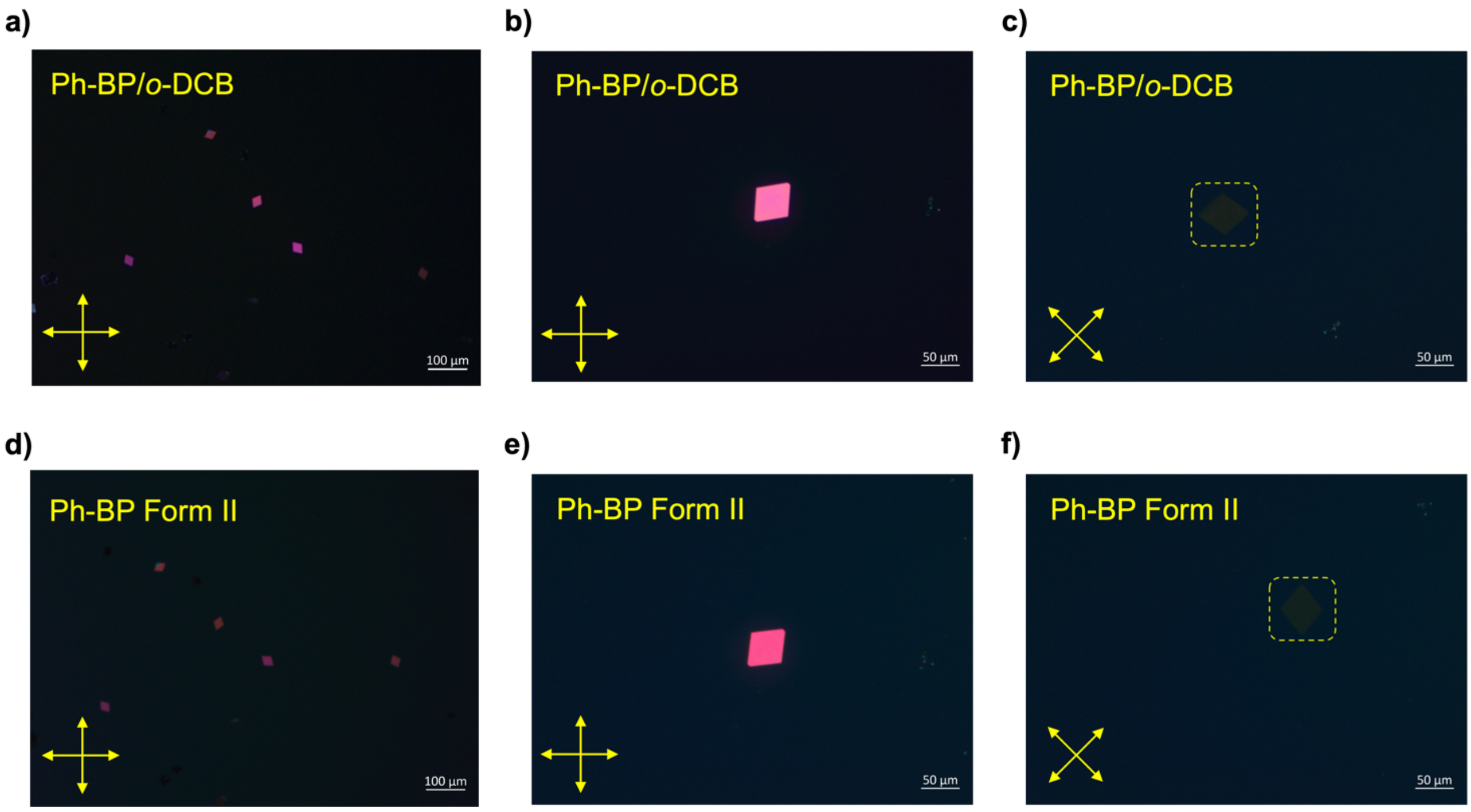

Figure S2. Polarized optical microscopy (POM) images of **Ph-BP**/*o*-DCB crystals before and after vacuum annealing at 180 °C under 6.7 × 10^{-2} Pa. a–c) Before annealing and d–f) after annealing. a, d) POM images showing multiple crystals, b, e) magnified images of a selected crystal, and c, f) corresponding images taken after rotating the substrate by 45° to achieve extinction. The crystals after vacuum annealing were confirmed to be **Ph-BP** Form II by out-of-plane X-ray diffraction (XRD) measurements.

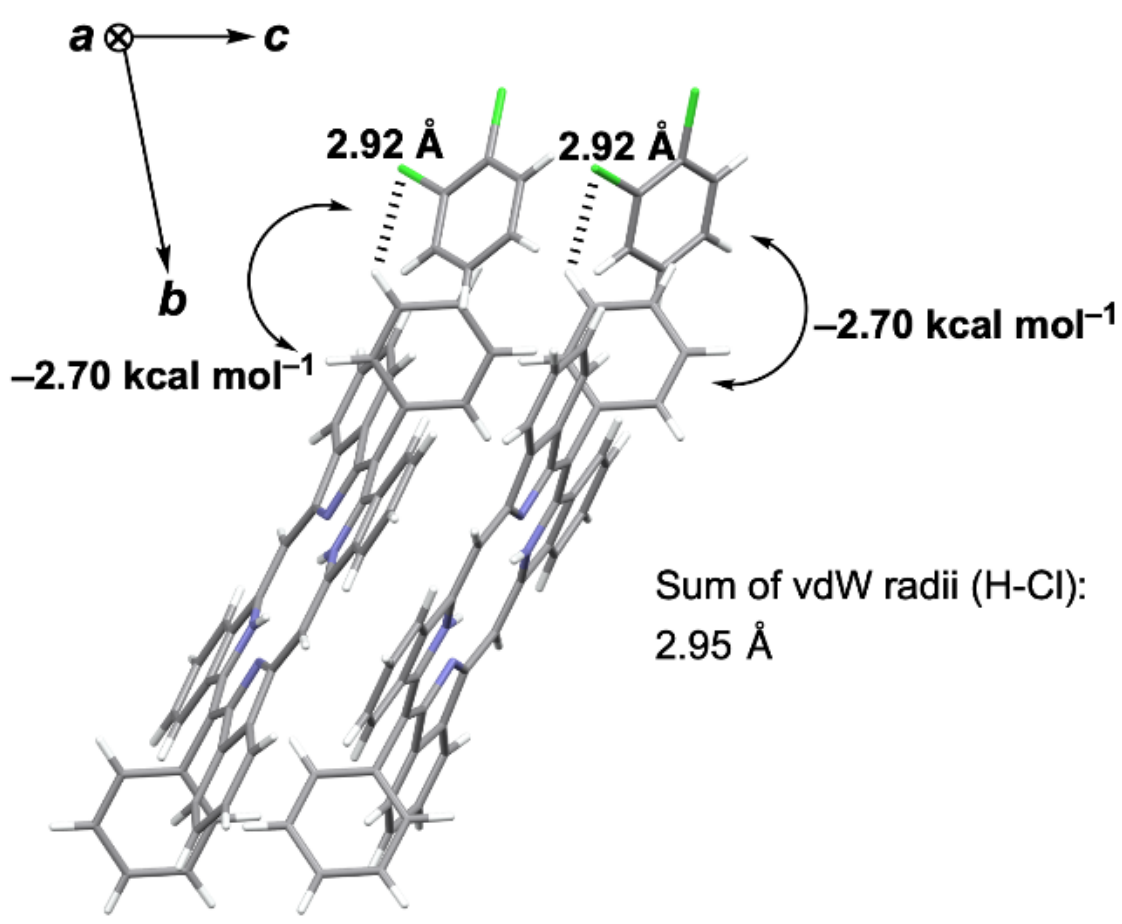

Figure S3. Intermolecular interactions between **Ph-BP** and *o*-DCB in the packing.

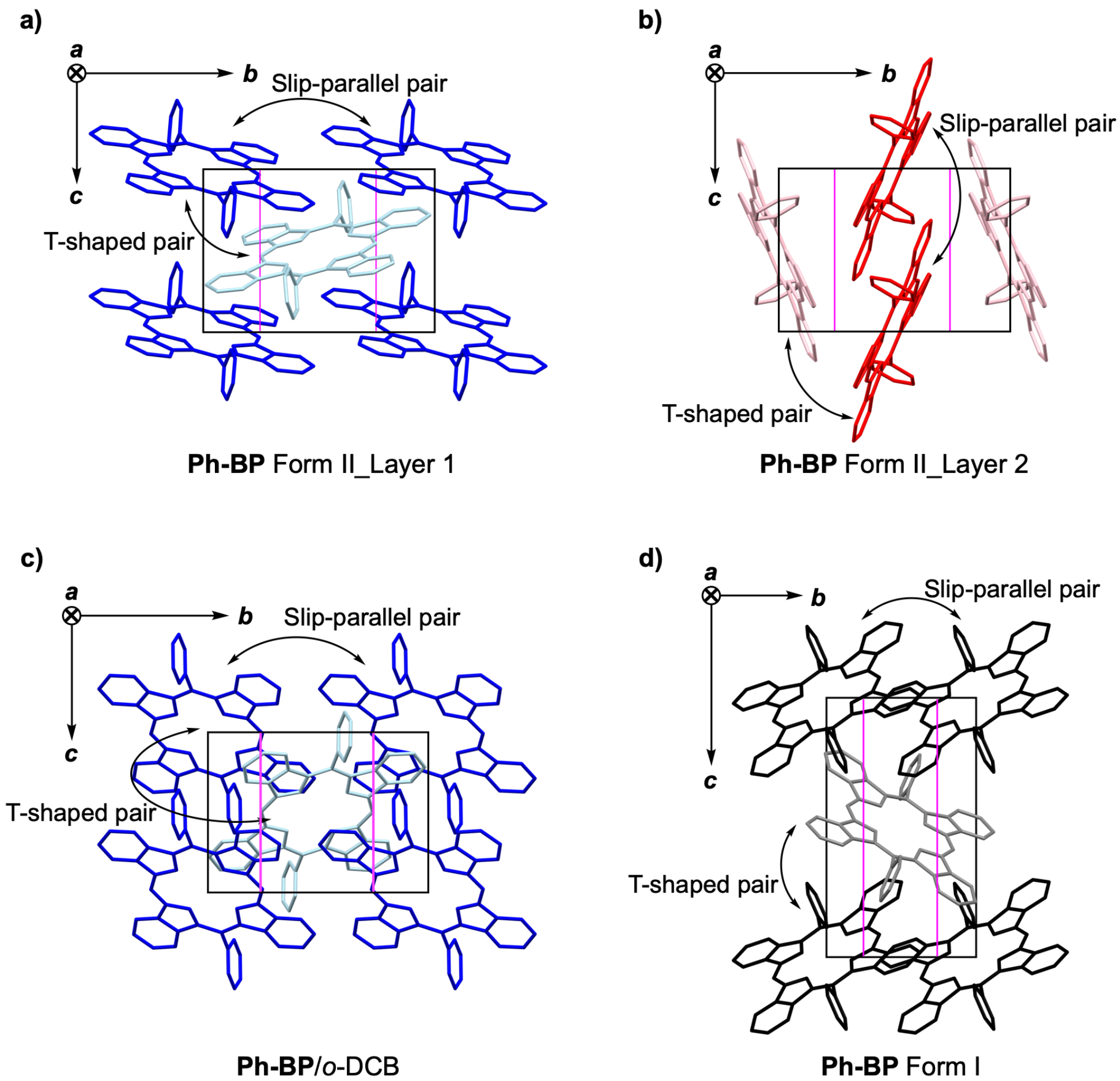

Figure S4. Molecular packing structures and glide-plane symmetry of a) Layer 1 and b) Layer 2 of **Ph-BP** Form II, c) **Ph-BP**/*o*-DCB, and d) **Ph-BP** Form I [2]. The glide planes are shown in pink, and molecules related by the glide-plane symmetry operation are distinguished by different color shades.

Table S2. Dihedral angles between the benzoporphyrin core planes and angles between the molecular long axes for the T-shaped and slip-parallel contacts in Layers 1 and 2 of **Ph-BP** Form II, **Ph-BP**/*o*-DCB, and **Ph-BP** Form I [2].

Packing Structure	Pair	Dihedral angle (°)	Long-axis angle (°)
**Ph-BP** Form II_Layer 1	T-shaped pair	23.90	40.17
	Slip-parallel pair	0	0
**Ph-BP** Form II_Layer 2	T-shaped pair	49.96	4.34
	Slip-parallel pair	0	0
**Ph-BP**/*o*-DCB	T-shaped pair	23.54	24.08
	Slip-parallel pair	0	0
**Ph-BP** Form I	T-shaped pair	49.31	20.04
	Slip-parallel pair	0	0

Table S3. SAPT calculated intermolecular interaction energies (kcal mol^{-1}).

Crystal structure	Pair	E_{total}	$E_{dispersion}$	$E_{electrostatic}$	$E_{induction}$	$E_{exchange}$
**Ph-BP**/*o*-DCB	Slip-parallel pair	–8.93	–11.12	–4.97	–1.00	8.15
	T-shaped pair	–31.50	–43.59	–10.14	–3.15	25.38
	With *o*-DCB	–2.70	–3.78	–1.44	–0.33	4.53
**Ph-BP** Form II	Slip-parallel pair_Layer 1	–8.43	–10.03	–4.03	–0.82	6.44
	T-shaped pair_Layer 1	–31.38	–43.56	–10.36	–3.14	25.68
	Slip-parallel pair_Layer 2	–16.37	–20.54	–4.37	–1.54	10.09
	T-shaped pair_Layer 2	–25.09	–36.76	–8.71	–2.39	22.77
	Interlayer_CH-π	–5.85	–8.37	–2.61	–0.68	5.81
**C_6F_5-BP**/*o*-DCB	Slip-parallel pair	–28.27	–37.19	–10.33	–2.81	22.06
	T-shaped pair	–8.50	–9.67	–3.26	–0.82	5.26
	With *o*-DCB	–2.54	–2.26	–1.25	–0.21	1.18
**C_6F_5-BP** bilayer	Slip-parallel pair	–27.81	–35.36	–9.69	–2.68	19.91
	T-shaped pair	–9.01	–11.17	–4.29	–1.05	7.49
	Interlayer_F-F	–4.13	–5.95	–1.49	–0.49	3.79
**Ph-BP** Form I[2]	Slip-parallel pair	–18.94	–24.88	–6.23	–2.20	14.38
	T-shaped pair	–29.97	–44.79	–10.62	–2.96	28.40
Pentacene	Slip-parallel pair	–9.67	–9.38	–3.66	–0.58	3.95
	T-shaped pair_1	–15.43	–19.42	–5.18	–1.58	10.75
	T-shaped pair_2	–15.26	–19.43	–5.30	–1.60	11.07

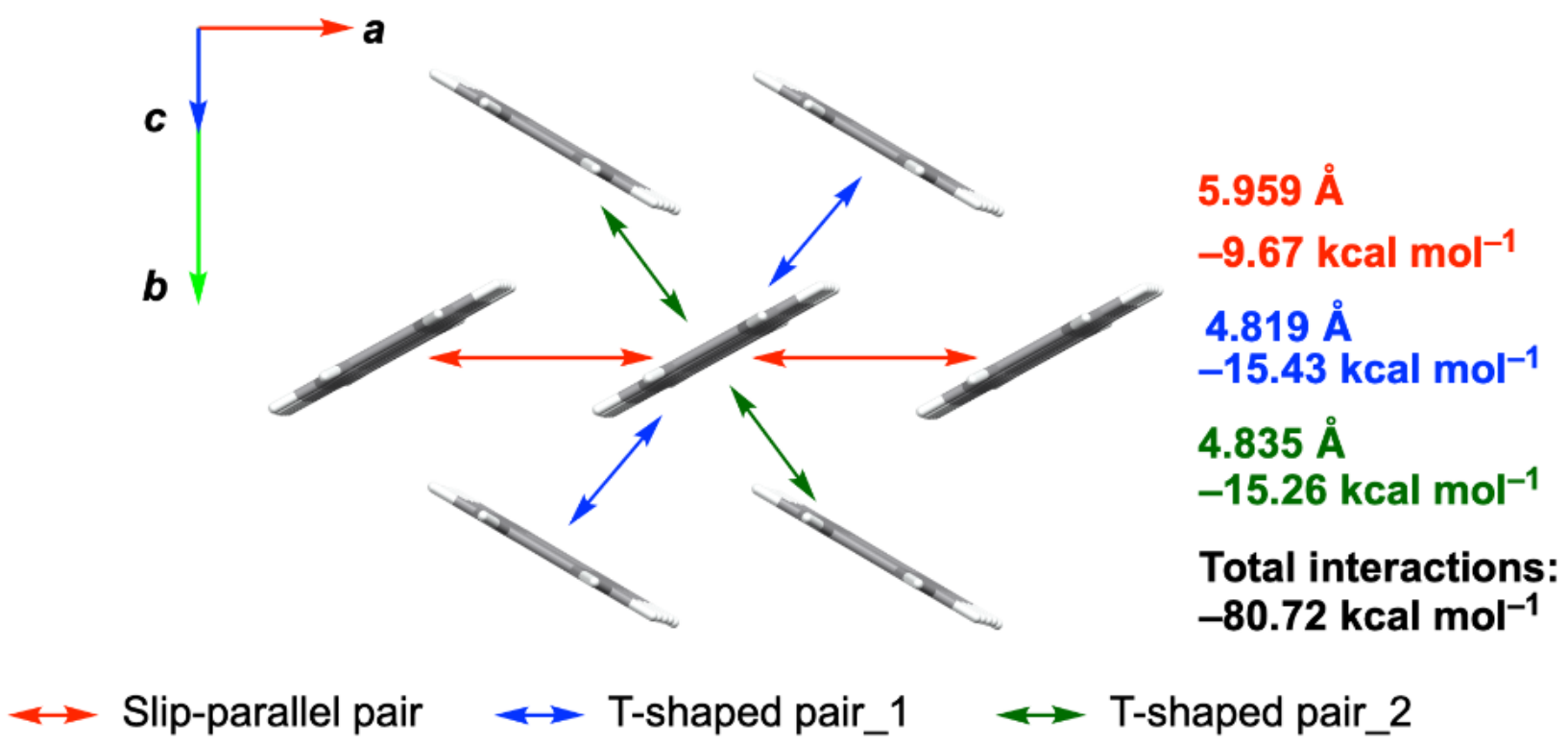

Figure S5. Centroid-to-centroid distances and intermolecular interaction energies in the packing of pentacene.

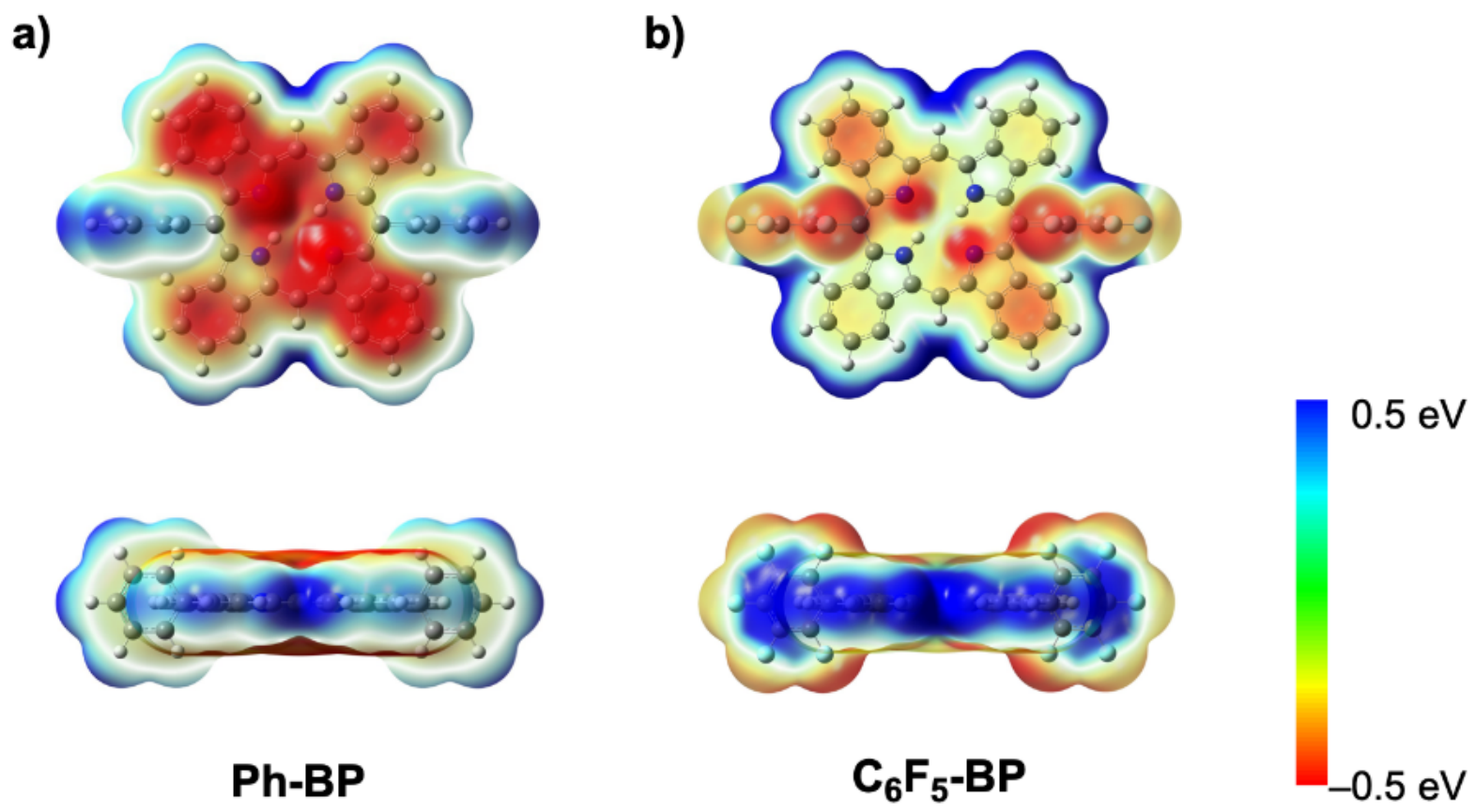

Figure S6. ESP distribution maps of a) **Ph-BP** and b) **C_6F_5-BP** shown in top and side views.

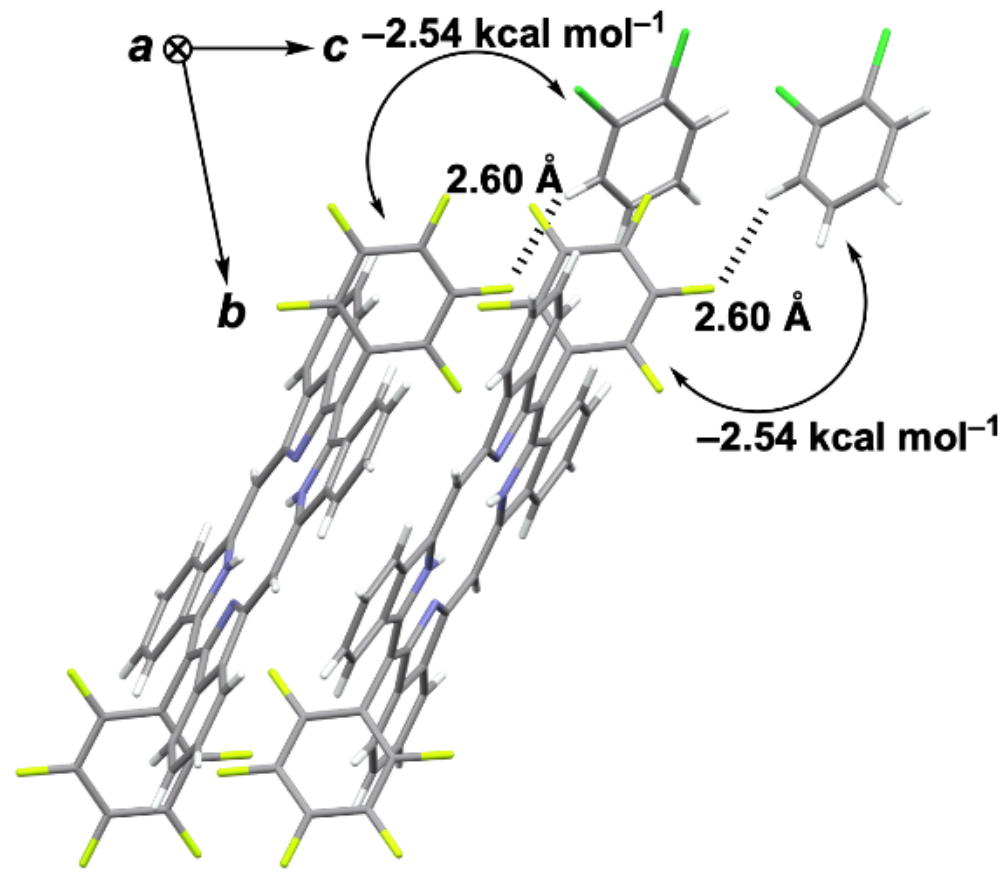

Figure S7. Short contacts and intermolecular interaction energies between **C_6F_5-BP** and *o*-DCB in the packing.

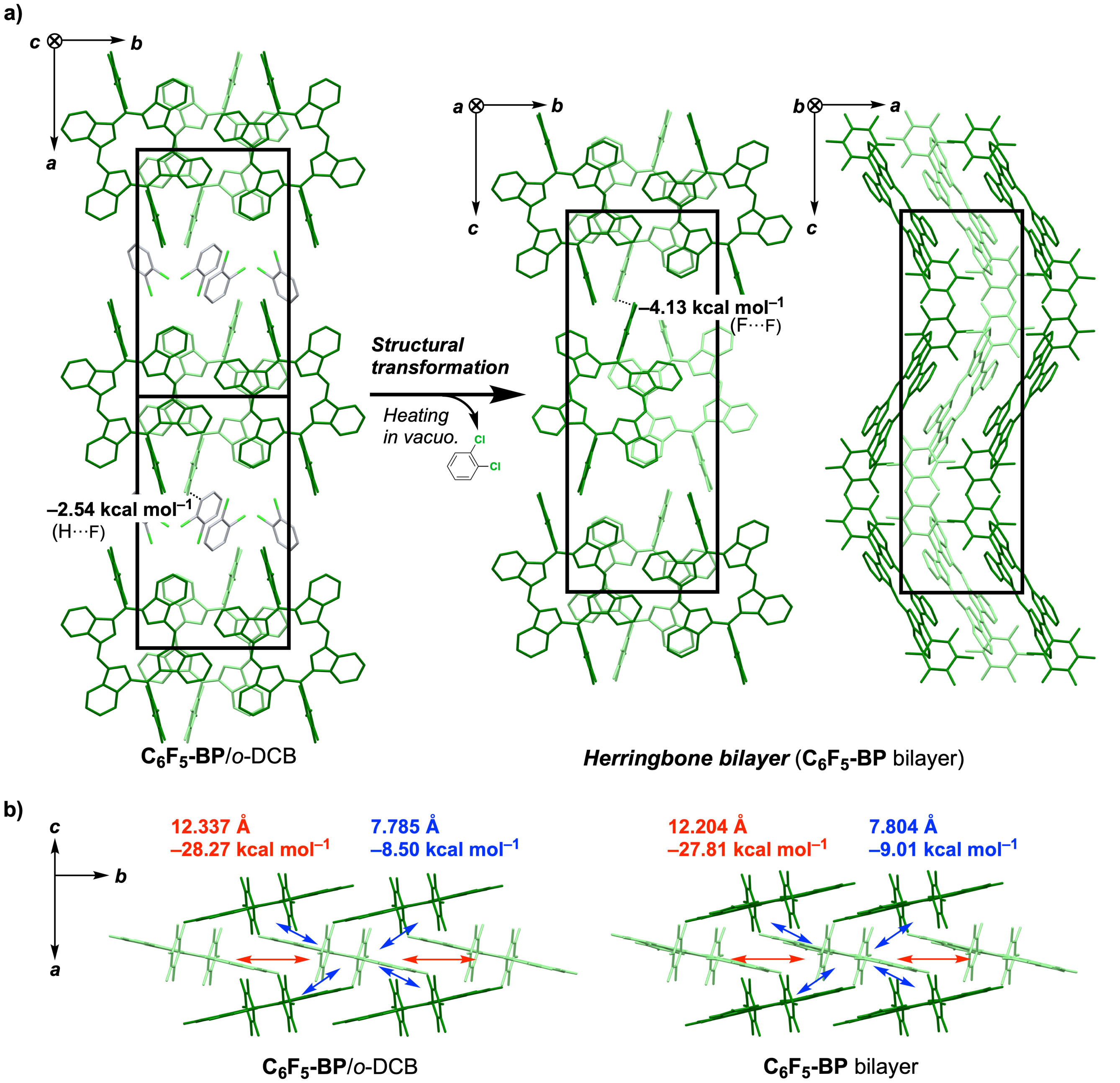

Figure S8. a) Schematic illustration of the structural transformation from **C_6F_5-BP**/*o*-DCB (CCDC: 2512998) to **C_6F_5-BP** bilayer (CCDC: 2512997). Hydrogen atoms are omitted for clarity. b) Centroid-to-centroid distances and intermolecular interaction energies in the packing of **C_6F_5-BP**/*o*-DCB (left) and **C_6F_5-BP** bilayer (right). The red and blue double-headed arrows denote slip-parallel and T-shaped pairs, respectively.

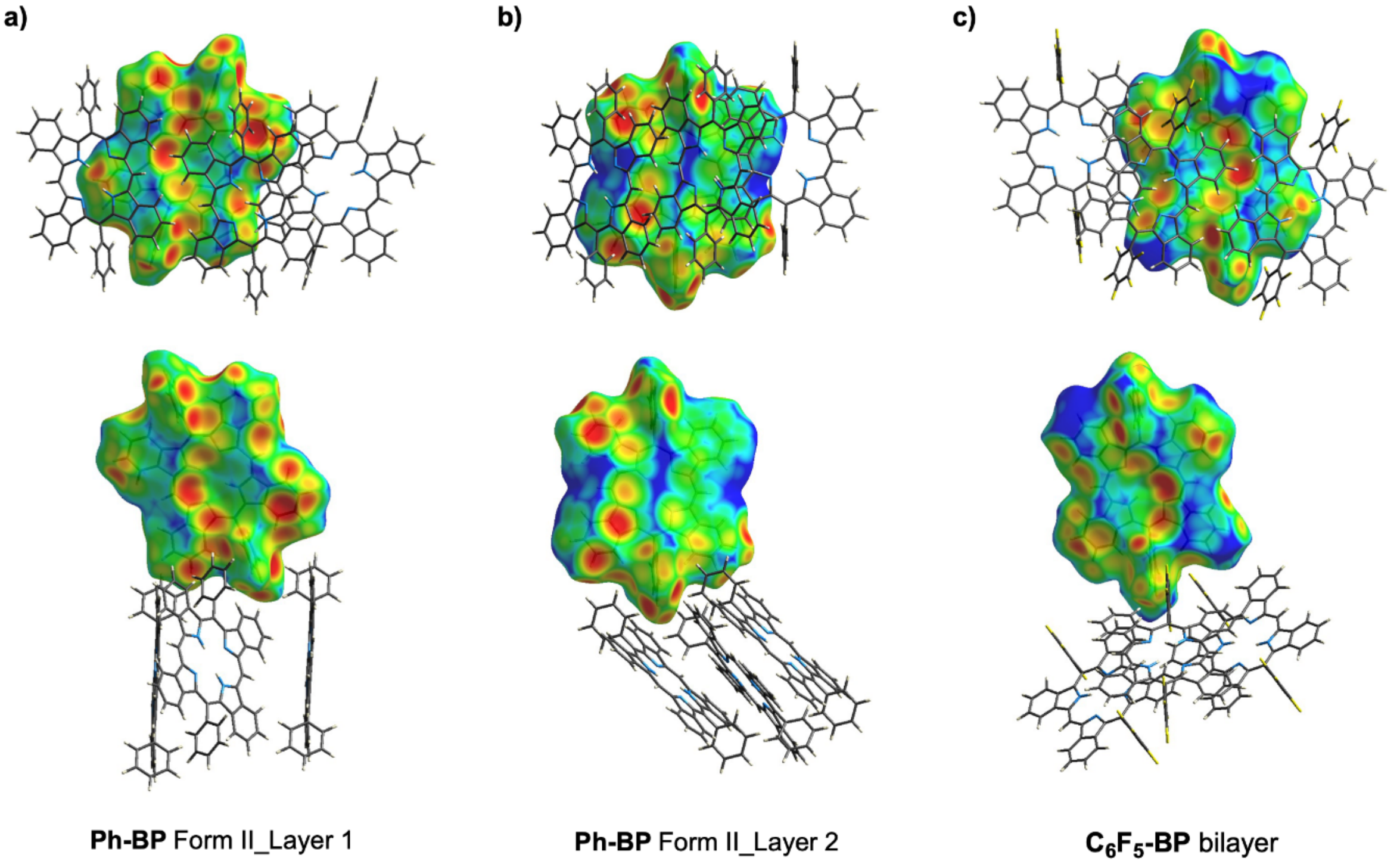

Figure S9. Hirshfeld surface analyses mapped with d_e (distance from the Hirshfeld surface to the nearest external nucleus) for the intralayer and interlayer packing structures of a) Layer 1 and b) Layer 2 of **Ph-BP** Form II, and c) **C_6F_5-BP** bilayer. The d_e color scale ranges from red at 0.12 Å to blue at 0.24 Å. The red depression spots on the Hirshfeld surfaces indicate close intermolecular contacts, including CH–π interactions.

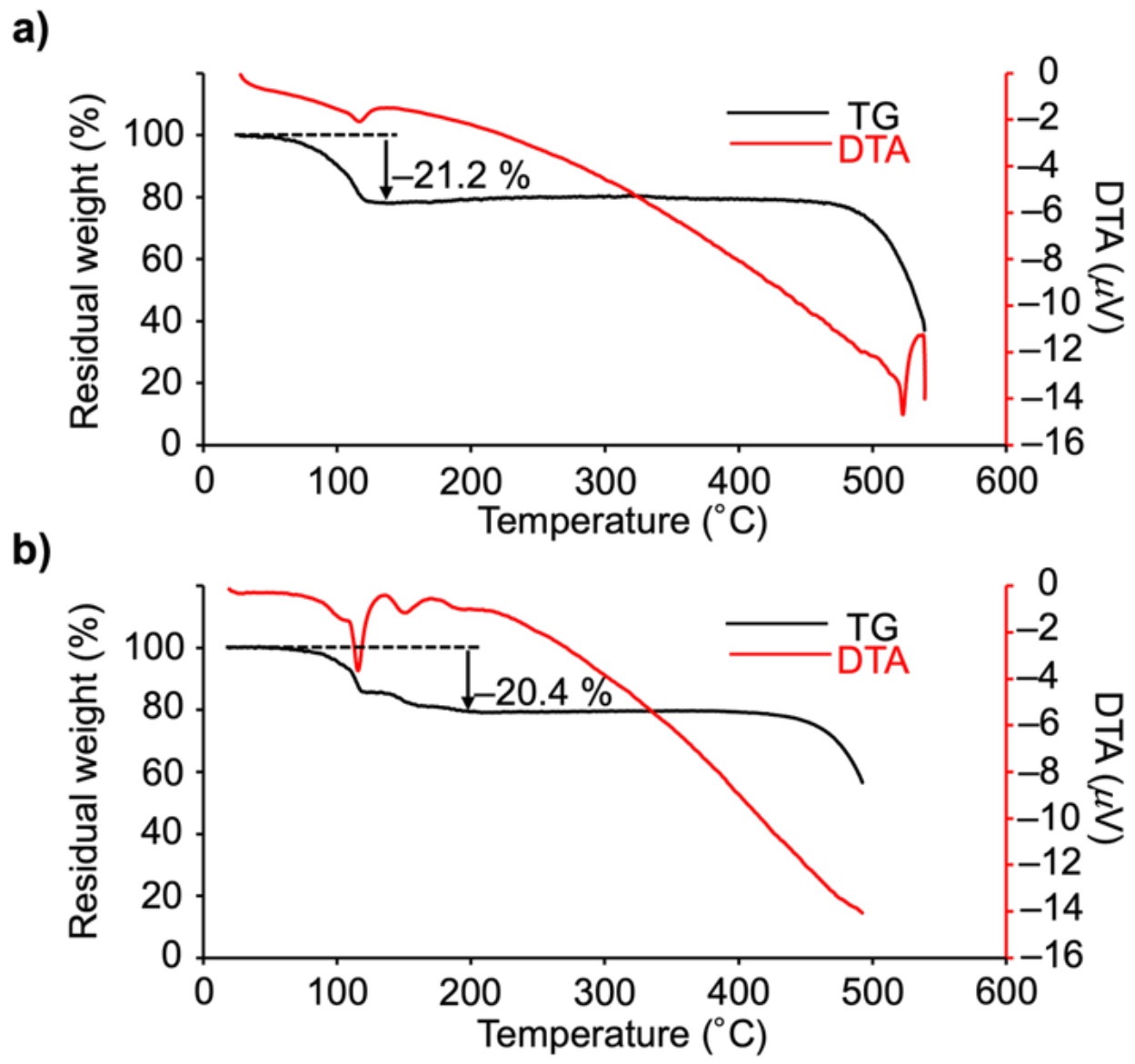

Figure S10. TG-DTA profiles of a) **Ph-BP**/*o*-DCB and b) **C_6F_5-BP**/*o*-DCB.

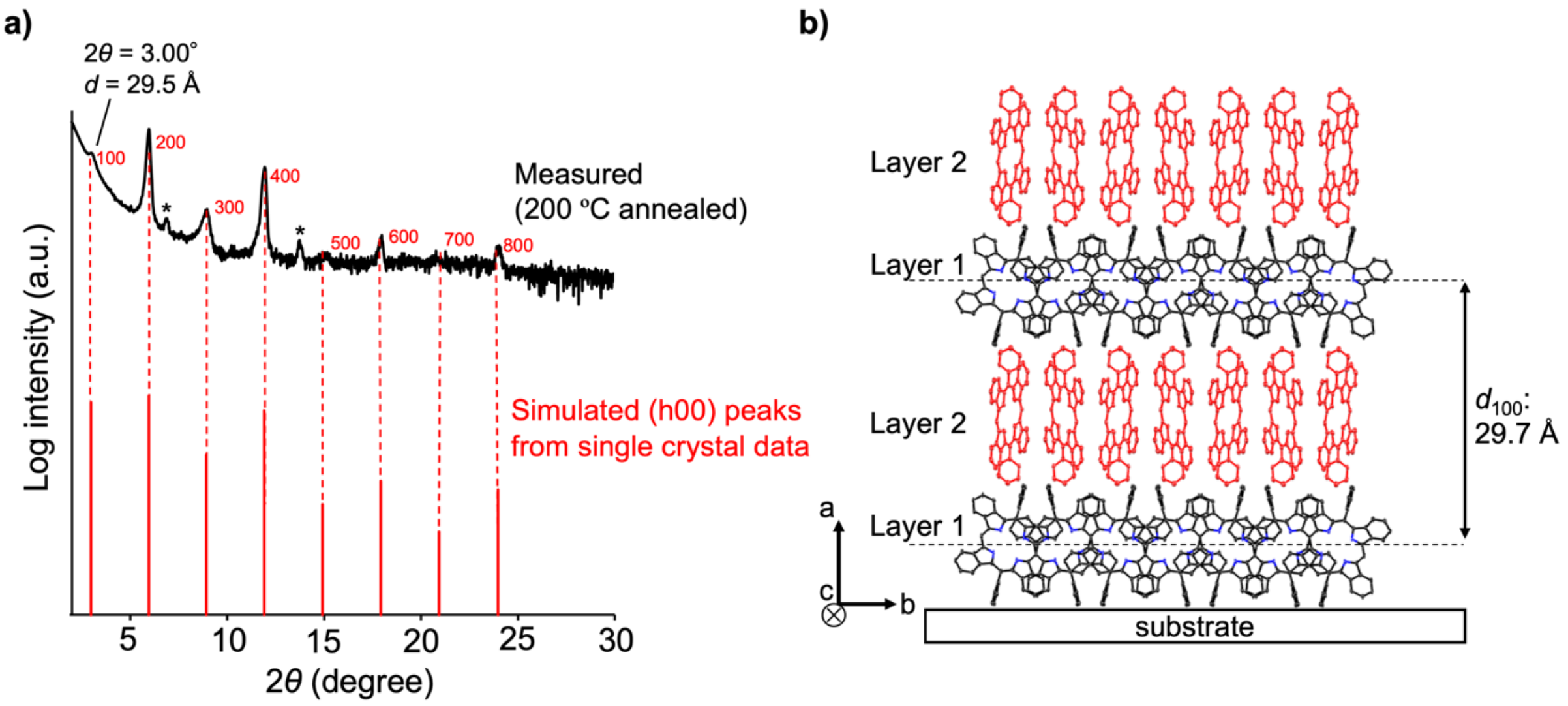

Figure S11. a) Out-of-plane XRD profiles of **Ph-BP**/*o*-DCB measured after heating at 200 °C, together with the simulated (h00) XRD pattern derived from the bulk single-crystal structure of the **Ph-BP** Form II. Peaks marked with an asterisk arise from a minor polymorph present in the crystals. b) Depiction of the layered structure of the **Ph-BP** Form II on the substrate.

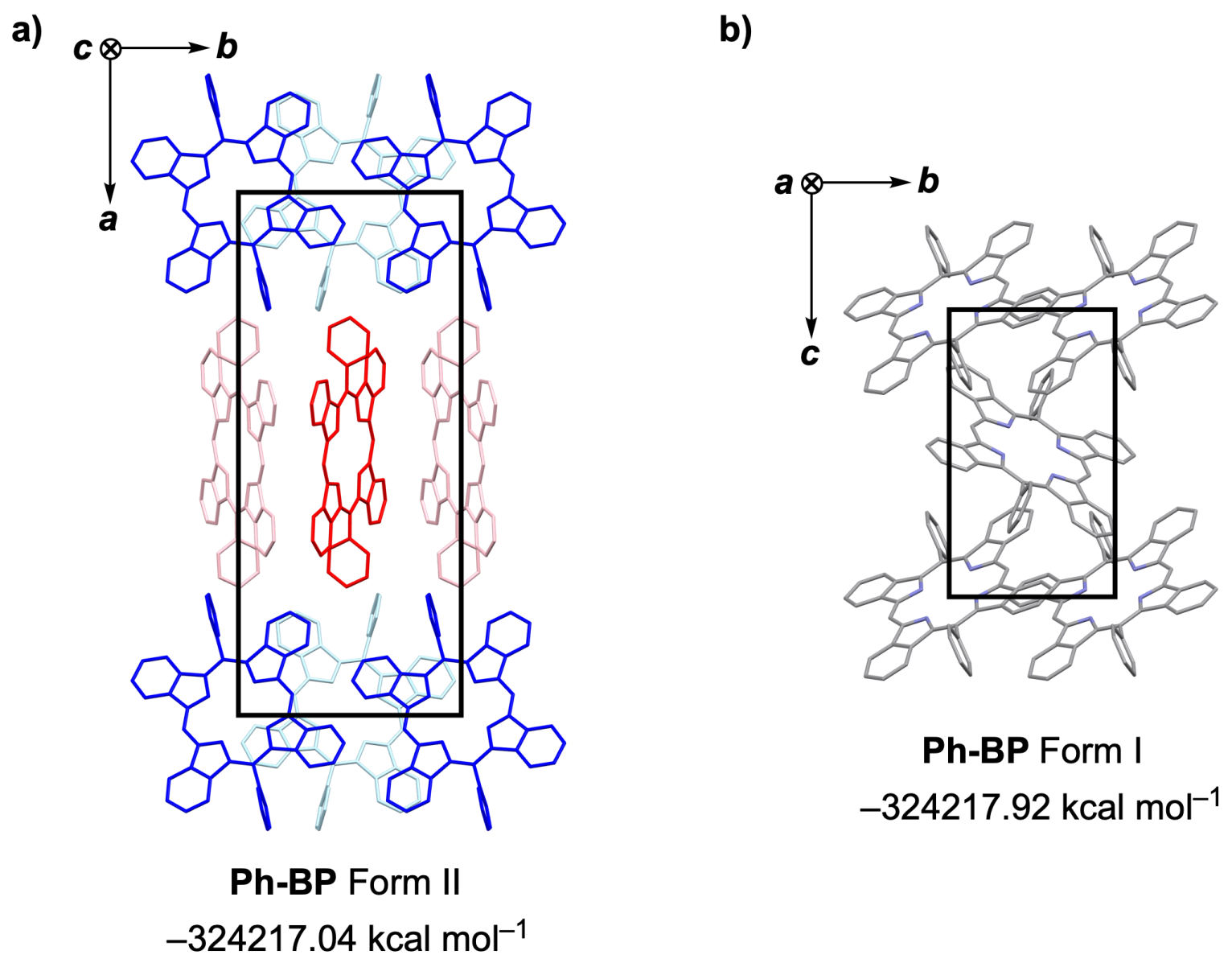

Figure S12. DFT calculated total energies for the crystal structure of a) **Ph-BP** Form II and b) **Ph-BP** Form I [2].

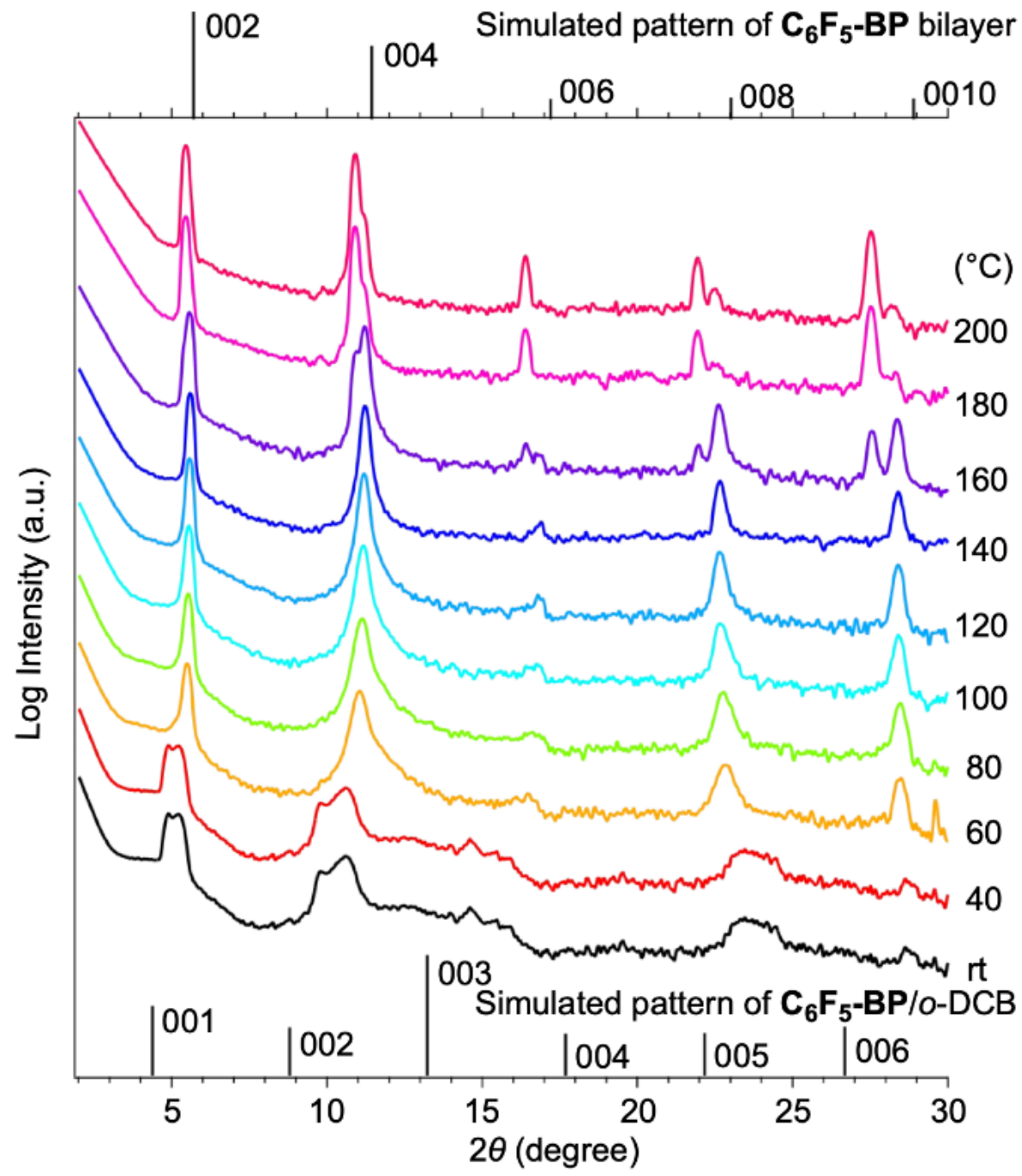

Figure S13. Out-of-plane XRD patterns of **C_6F_5-BP** crystals grown on Si/SiO_2 substrates during stepwise annealing from 40 to 200 °C.

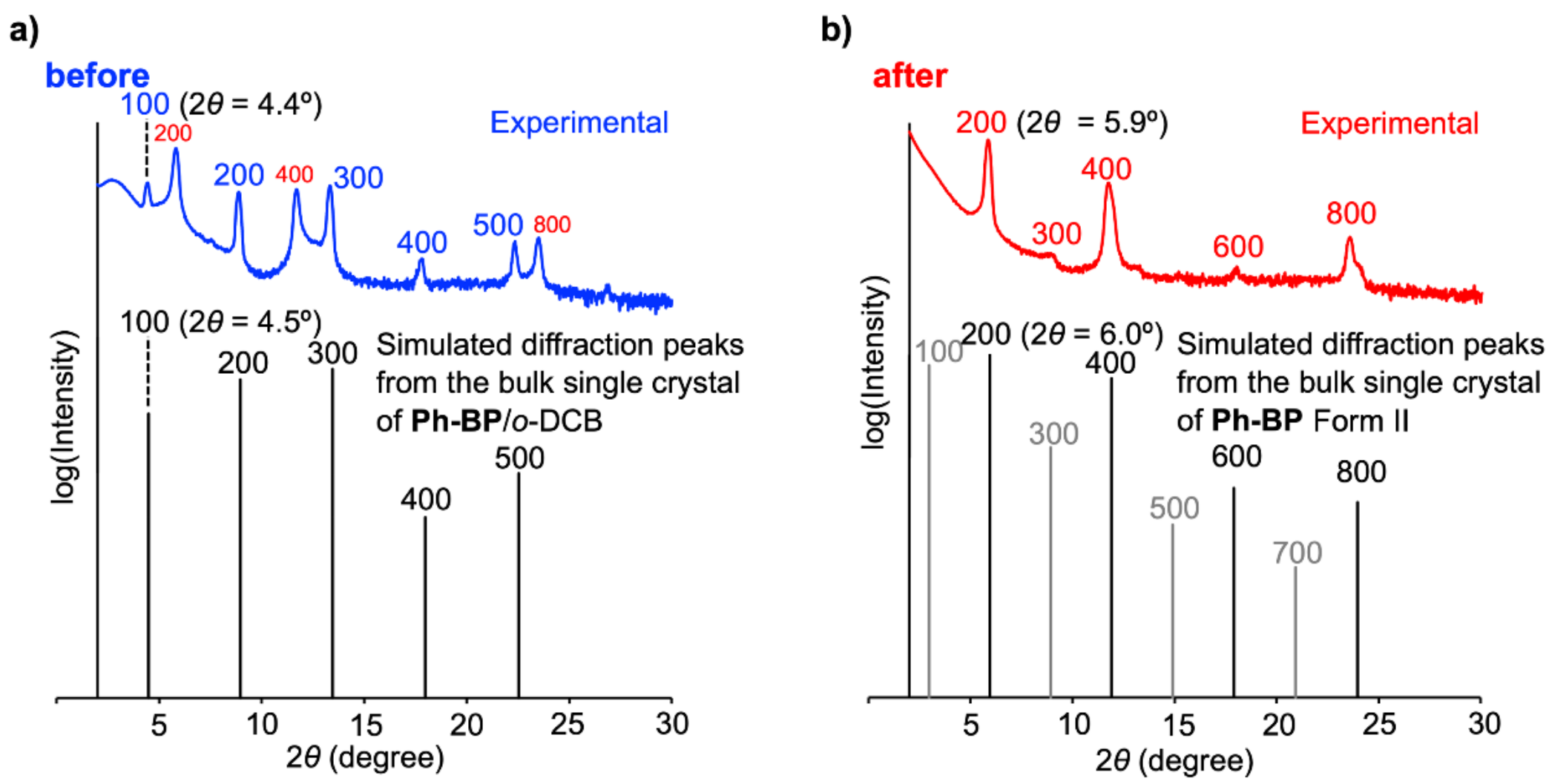

Figure S14. Out-of-plane XRD profiles of single crystals of **Ph-BP** grown on Si/SiO_2 substrate a) before and b) after thermal evaporation of Au electrodes, together with simulated peak positions derived from the bulk single-crystal X-ray diffraction data.

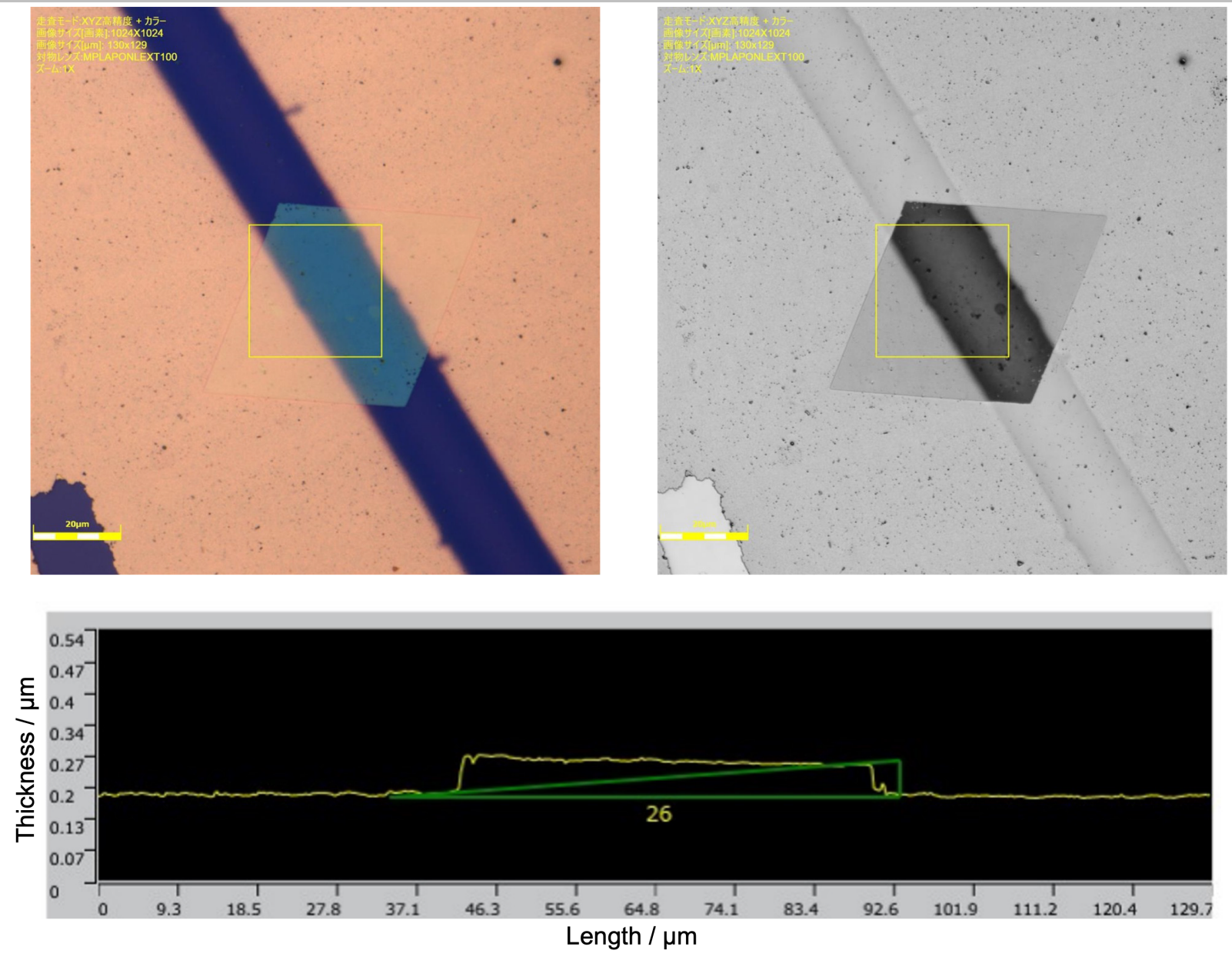

Figure S15. Thickness profile of a single crystal of **Ph-BP** grown on a Si/SiO_2 substrate, obtained by confocal laser scanning microscopy.

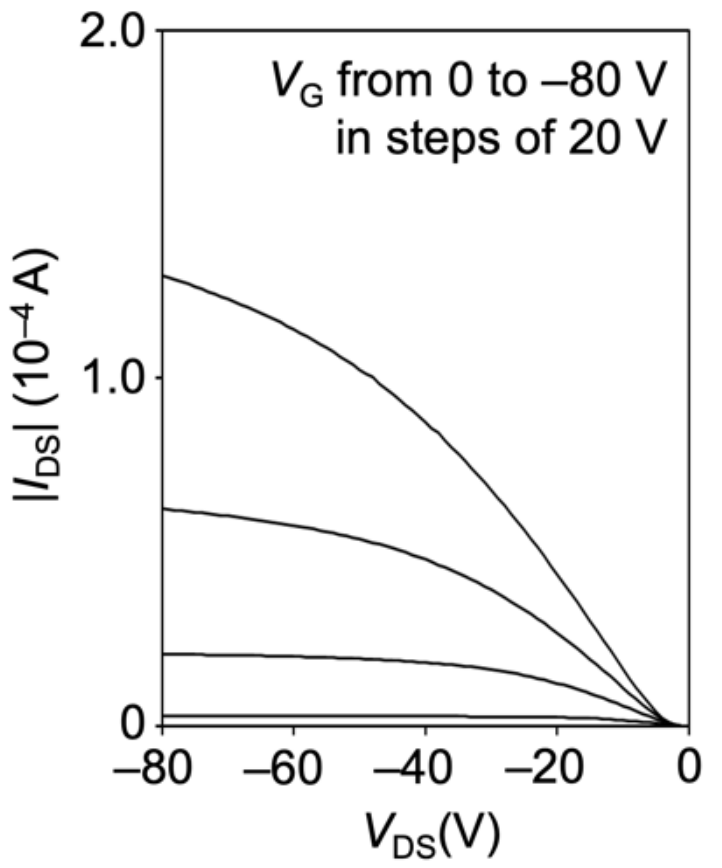

Figure S16. Output characteristics of a representative SC-OFET based on **Ph-BP**.

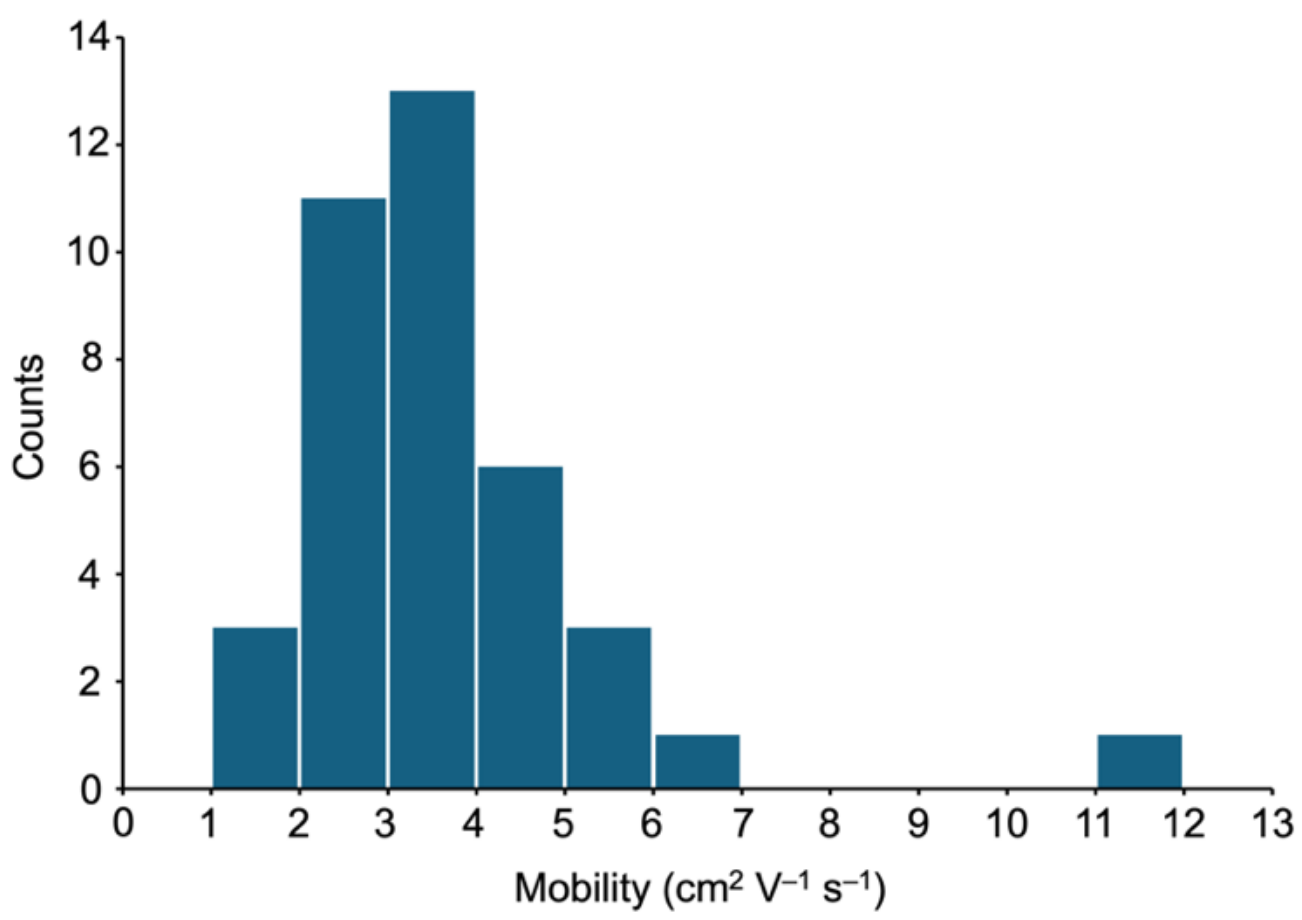

Figure S17. Histogram of the field-effect hole mobilities (μ_h) extracted from the transfer characteristics in the saturation regime for all measured **Ph-BP** single-crystal OFET devices (n = 36, including the exceptional device).

Table S4. SC-OFET characteristics of **Ph-BP** crystals with electrodes aligned along the *b*-axis.

Device	μ_h [a] ($cm^2 V^{-1} s^{-1}$)	V_{th} (V)	I_{on} (A)	I_{off} (A)	I_{on} / I_{off}	$\mu_{h,Y}$ [b] ($cm^2 V^{-1} s^{-1}$)	$V_{th,Y}$ [c] (V)
1	3.1	–13.4	-9.78×10^{-5}	-4.94×10^{-8}	2.0×10^{3}	–	–
2	2.7	–14.1	-1.24×10^{-4}	-1.07×10^{-7}	1.2×10^{3}	–	–
3	3.9	–23.8	-1.74×10^{-4}	-6.74×10^{-9}	2.6×10^{4}	–	–
4	3.0	–24.0	-1.27×10^{-4}	-1.10×10^{-8}	1.2×10^{4}	–	–
5	2.7	–13.3	-1.35×10^{-4}	-4.09×10^{-8}	3.3×10^{3}	–	–
6	3.2	–15.9	-1.39×10^{-4}	-3.78×10^{-8}	3.7×10^{3}	–	–
7	3.2	–18.2	-6.05×10^{-5}	-1.65×10^{-8}	3.7×10^{3}	–	–
8	11.3	–12.9	-3.04×10^{-4}	-1.34×10^{-6}	2.3×10^{2}	–	–
9	3.7	–3.4	-1.52×10^{-4}	-2.70×10^{-7}	5.6×10^{2}	–	–
10	5.5	–21.8	-2.88×10^{-4}	-1.19×10^{-8}	2.4×10^{4}	–	–
11	2.6	–16.2	-9.14×10^{-5}	-2.69×10^{-8}	3.4×10^{3}	–	–
12	4.1	–23.0	-8.35×10^{-5}	-1.71×10^{-9}	4.9×10^{4}	–	–
13	3.3	–13.7	-1.53×10^{-4}	-4.54×10^{-8}	3.4×10^{3}	–	–
14	2.5	–13.7	-2.12×10^{-4}	-4.92×10^{-8}	4.3×10^{3}	–	–
15	6.0	–22.9	-2.85×10^{-4}	-2.32×10^{-8}	1.2×10^{4}	–	–
16	3.4	–15.8	-2.56×10^{-4}	-2.47×10^{-9}	1.0×10^{5}	–	–
17	3.2	–5.3	-2.04×10^{-4}	-6.81×10^{-7}	3.0×10^{2}	–	–
18	3.7	–13.2	-1.22×10^{-4}	-1.81×10^{-8}	6.7×10^{3}	–	–
19	2.1	–18.5	-7.84×10^{-5}	-4.84×10^{-9}	1.6×10^{4}	–	–
20	3.8	–14.8	-2.16×10^{-4}	-2.84×10^{-8}	7.6×10^{3}	–	–
21	3.5	–13.6	-1.33×10^{-4}	-2.85×10^{-8}	4.7×10^{3}	–	–
22	3.2	–15.1	-1.49×10^{-4}	-5.63×10^{-8}	2.6×10^{3}	–	–
23	4.1	–24.9	-7.01×10^{-5}	-1.03×10^{-8}	6.8×10^{3}	–	–
24	2.6	–12.7	-1.32×10^{-4}	-8.50×10^{-8}	1.6×10^{3}	–	–
25	5.7	–27.1	-1.51×10^{-4}	-1.16×10^{-8}	1.3×10^{4}	–	–
26	3.0	–14.2	-1.12×10^{-4}	-1.07×10^{-8}	1.1×10^{4}	–	–
27	4.3	–31.3	-1.50×10^{-4}	-1.24×10^{-9}	1.2×10^{5}	1.4	–20.8
28	6.6	–12.8	-1.36×10^{-4}	-6.26×10^{-7}	2.2×10^{2}	5.2	–15.8
29	1.8	–14.2	-8.62×10^{-5}	-1.43×10^{-7}	6.0×10^{2}	1.6	–12.3
30	2.2	–9.4	-1.26×10^{-4}	-6.48×10^{-8}	1.9×10^{3}	1.0	–9.6
31	1.7	–8.4	-1.42×10^{-4}	-1.99×10^{-7}	7.1×10^{2}	1.0	–12.8
32	4.6	–35.1	-9.05×10^{-5}	-1.27×10^{-9}	7.1×10^{4}	5.2	–21.4
33	4.1	–19.9	-2.10×10^{-4}	-3.20×10^{-8}	6.6×10^{3}	2.7	–6.3
34	1.8	–8.1	-9.37×10^{-5}	-2.89×10^{-8}	3.2×10^{3}	2.0	–10.6
35	2.6	–7.7	-8.72×10^{-5}	-1.00×10^{-8}	8.6×10^{3}	3.0	–2.6
36	4.0	–8.2	-1.76×10^{-4}	-1.87×10^{-7}	9.4×10^{2}	2.7	–8.0

[a] The average mobility excluding the exceptionally high value of 11.3 $cm^2 V^{-1} s^{-1}$ was 3.5 ± 1.2 $cm^2 V^{-1} s^{-1}$ (mean ± standard deviation). [b] The mobility values were determined using the Y-function method. The average mobility was 2.6 ± 1.5 $cm^2 V^{-1} s^{-1}$ (mean ± standard deviation). [c] The threshold voltage was determined using the Y-function method.

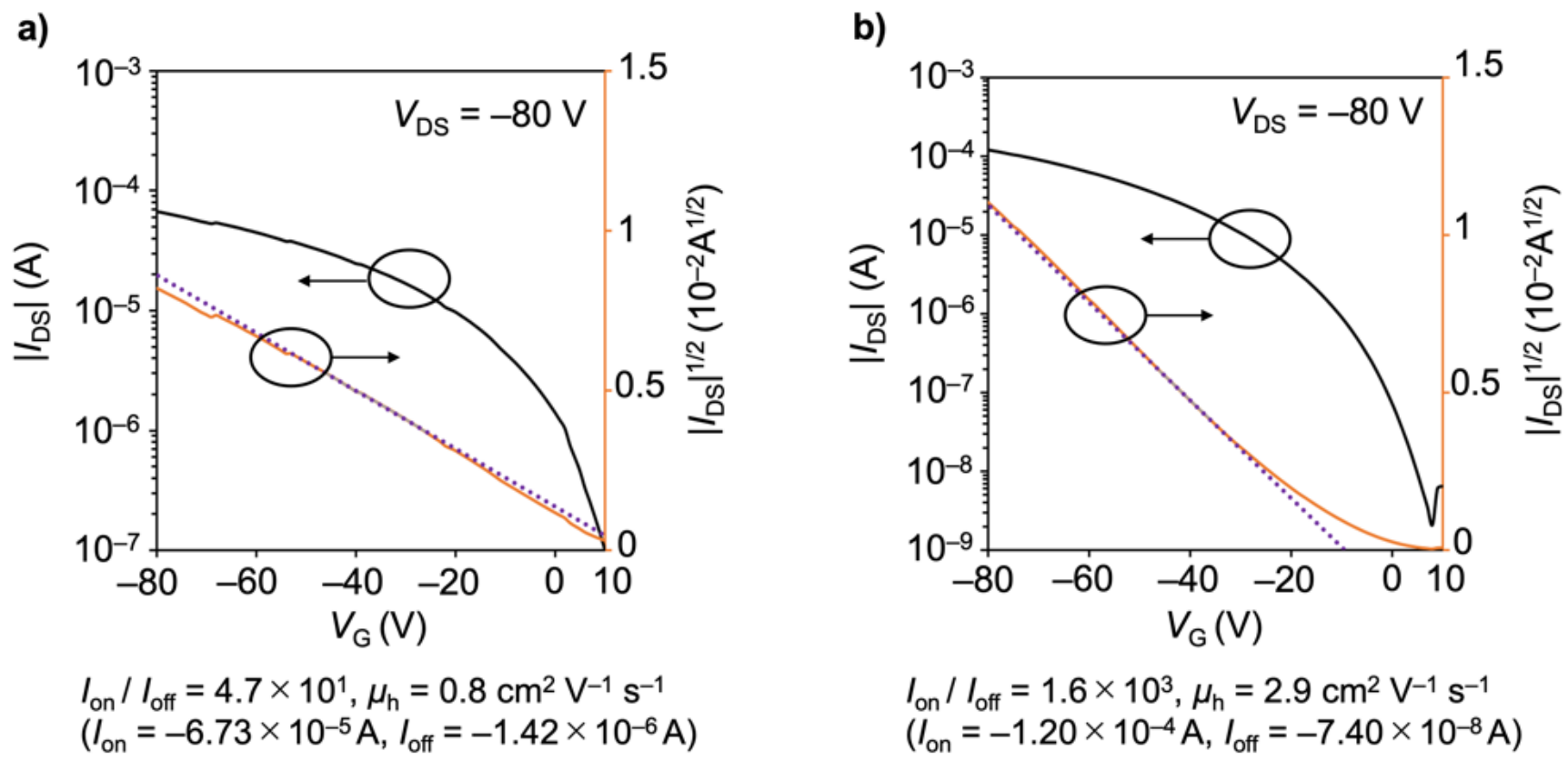

Figure S18. Transfer characteristics of the device exhibiting a relatively high off-state current, measured under a) ambient and b) vacuum conditions.

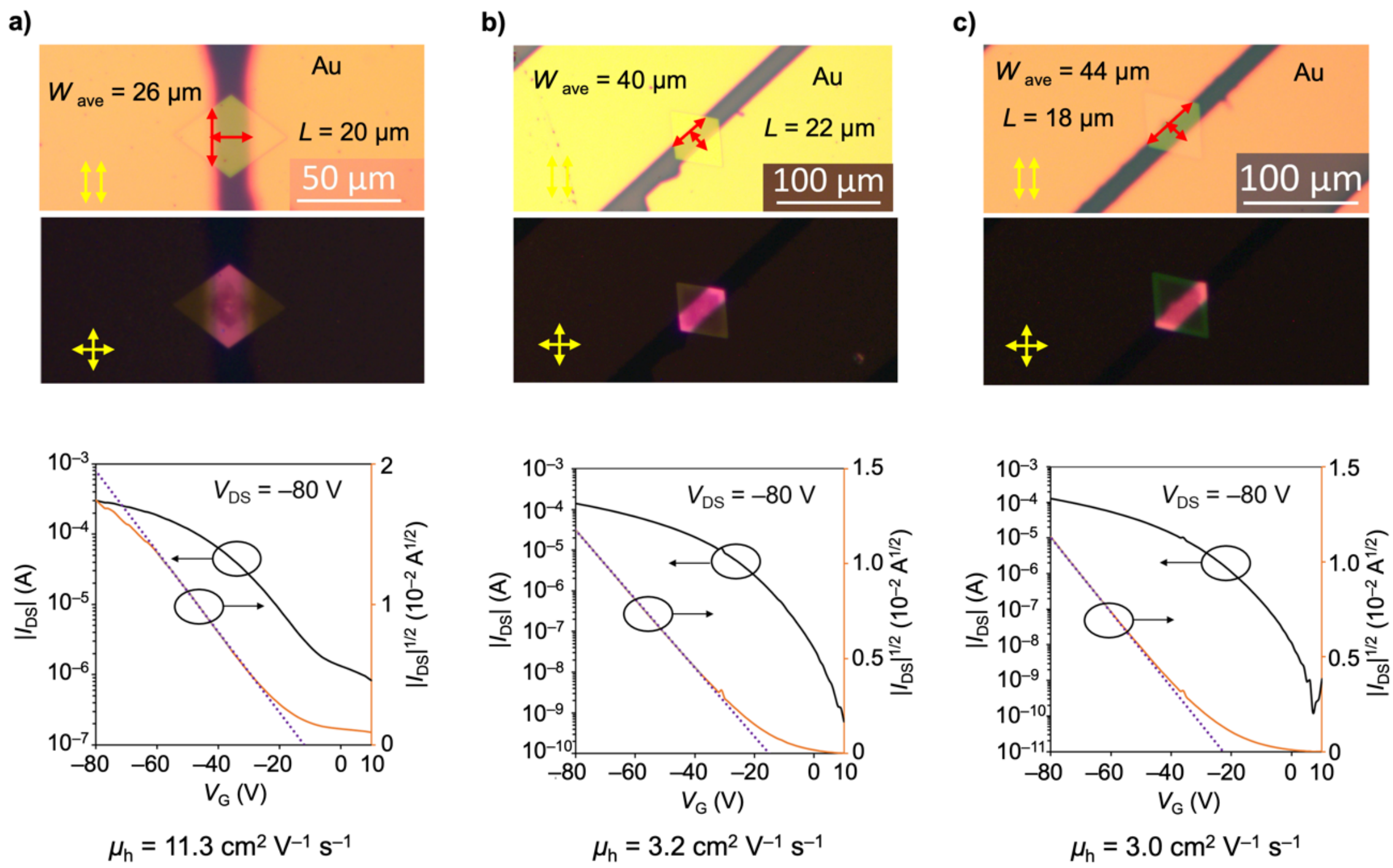

Figure S19. Optical micrographs (top), polarized optical micrographs (middle), and transfer characteristics (bottom) of **Ph-BP** SC-OFET devices exhibiting different field-effect mobilities.

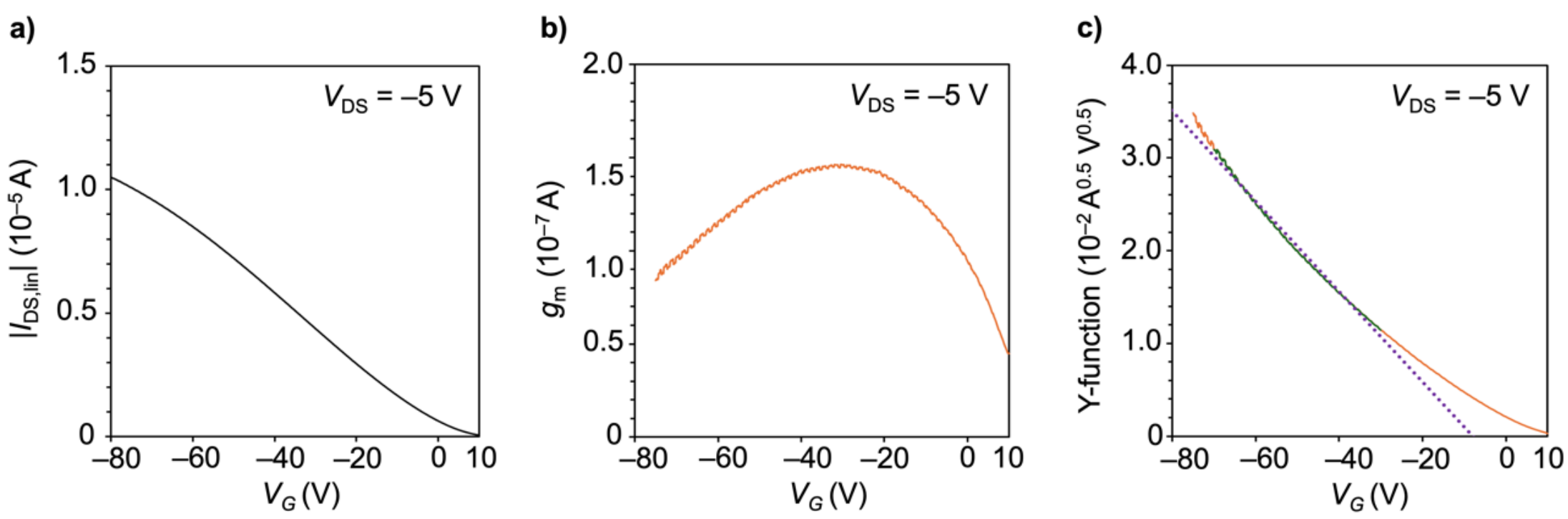

Figure S20. a) Transfer characteristics, b) transconductance (g_m) versus gate voltage, and c) Y-function plot of a representative **Ph-BP** SC-OFET measured at V_{DS} = –5 V.

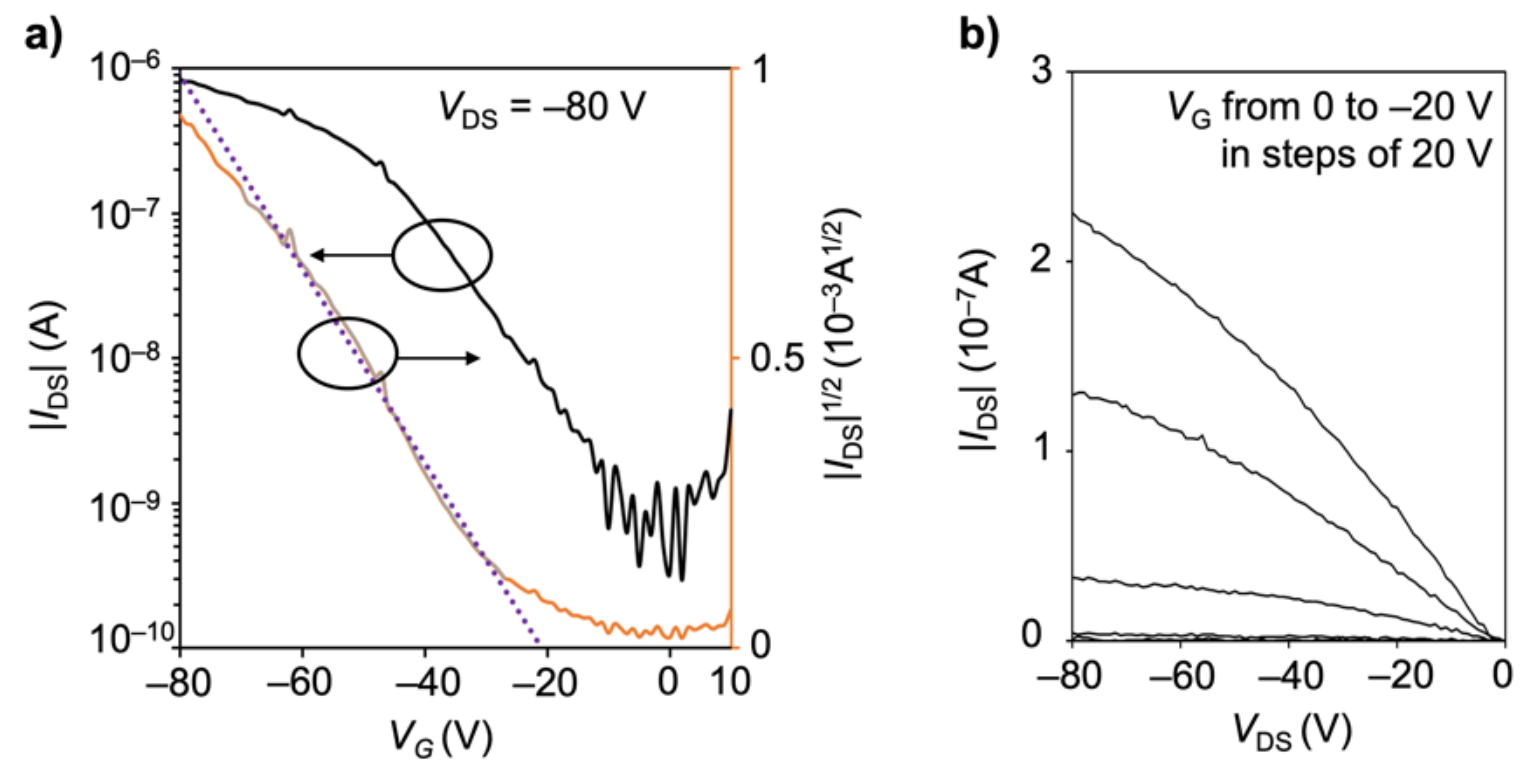

Figure S21. a) Transfer and b) output characteristics of **C_6F_5-BP**.

Table S5. SC-OFET characteristics of **C_6F_5-BP** crystals.

Device	μ_h (cm^2 V^{-1} s^{-1})	V_{th} (V)	I_{on} (A)	I_{off} (A)	I_{on} / I_{off}
1	3.0×10^{-2}	–20.9	-8.46×10^{-7}	-3.26×10^{-10}	2.6×10^{3}
2	1.3×10^{-2}	–26.9	-2.49×10^{-6}	-1.57×10^{-8}	1.6×10^{2}

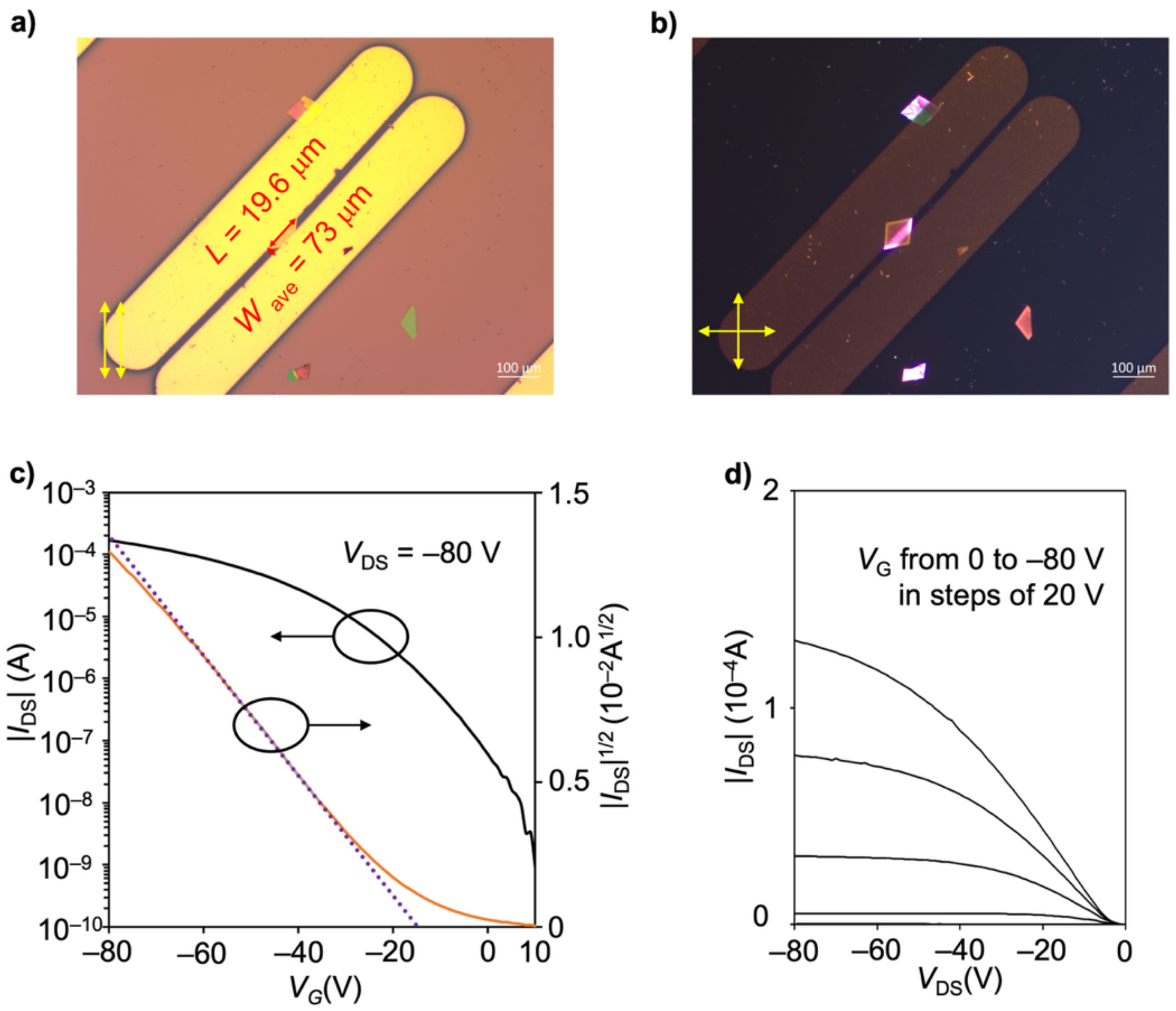

Figure S22. a) Optical micrograph, b) polarized optical micrograph, c) transfer, and d) output characteristics of the SC-OFET based on **Ph-BP** with electrodes aligned along the *c*-axis.

Table S6. SC-OFET characteristics of **Ph-BP** crystals with electrodes aligned along the *c*-axis.

Device	μ_h (cm^2 V^{-1} s^{-1})	V_{th} (V)	I_{on} (A)	I_{off} (A)	I_{on} / I_{off}
1	1.6	–13.4	-1.09×10^{-4}	-4.64×10^{-8}	2.3×10^{3}
2	0.7	–5.2	-9.63×10^{-5}	-2.01×10^{-7}	4.8×10^{2}
3	1.6	–8.7	-1.04×10^{-4}	-9.14×10^{-8}	1.1×10^{3}
4	2.0	–14.8	-1.68×10^{-4}	-5.96×10^{-8}	2.8×10^{3}

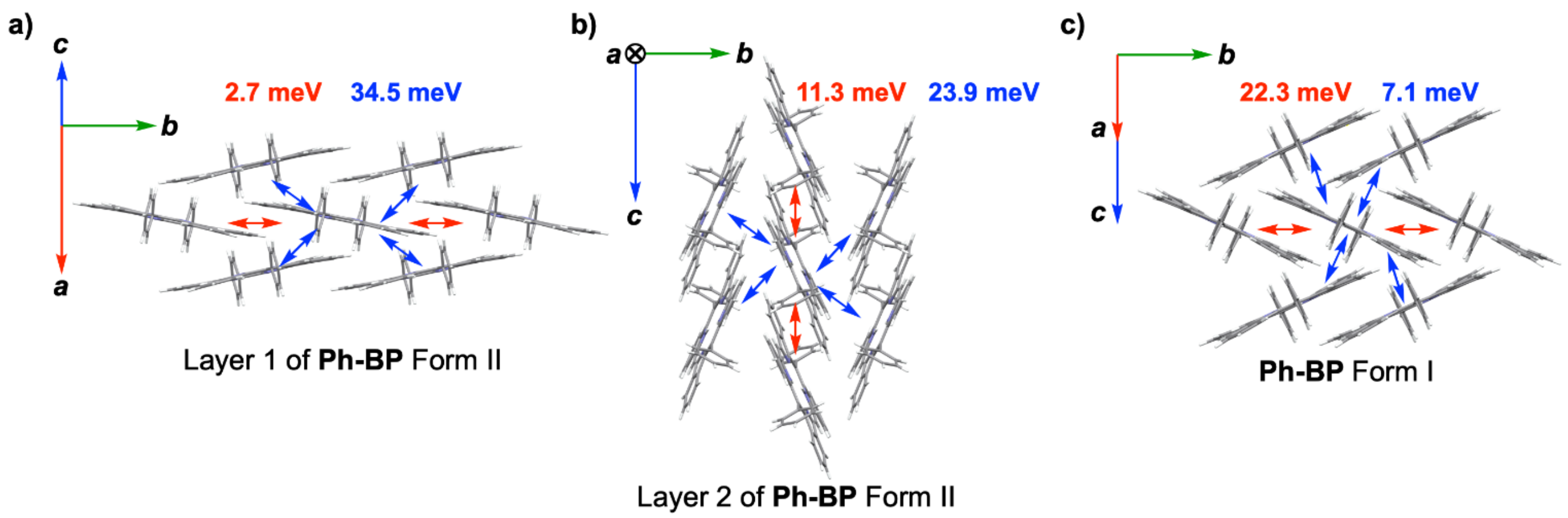

Figure S23. Transfer integrals in the packing of a) Layer 1, b) Layer 2 of **Ph-BP** Form II, and c) **Ph-BP** Form I [2].

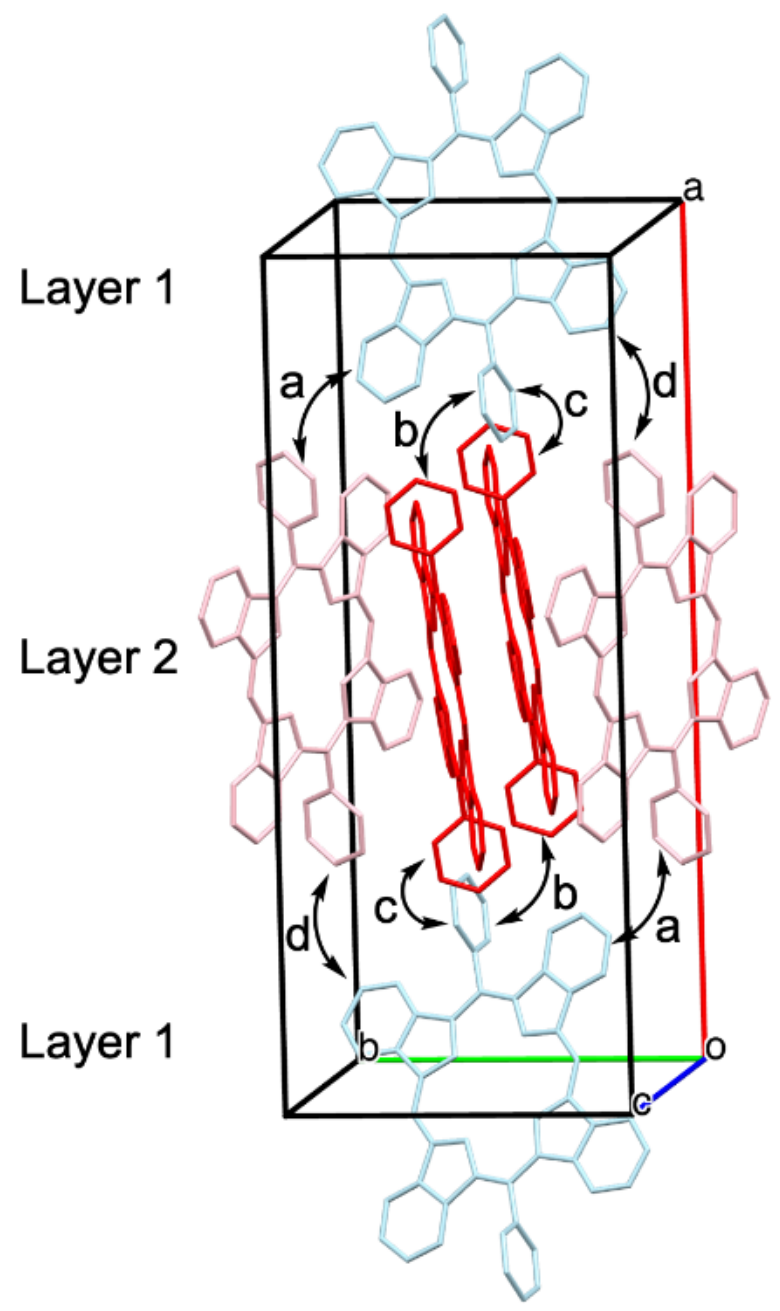

Pair	t_{hole} (meV)
a	0.74
b	0.78
c	0.21
d	0.14

Figure S24. Interlayer transfer integrals in the **Ph-BP** Form II.

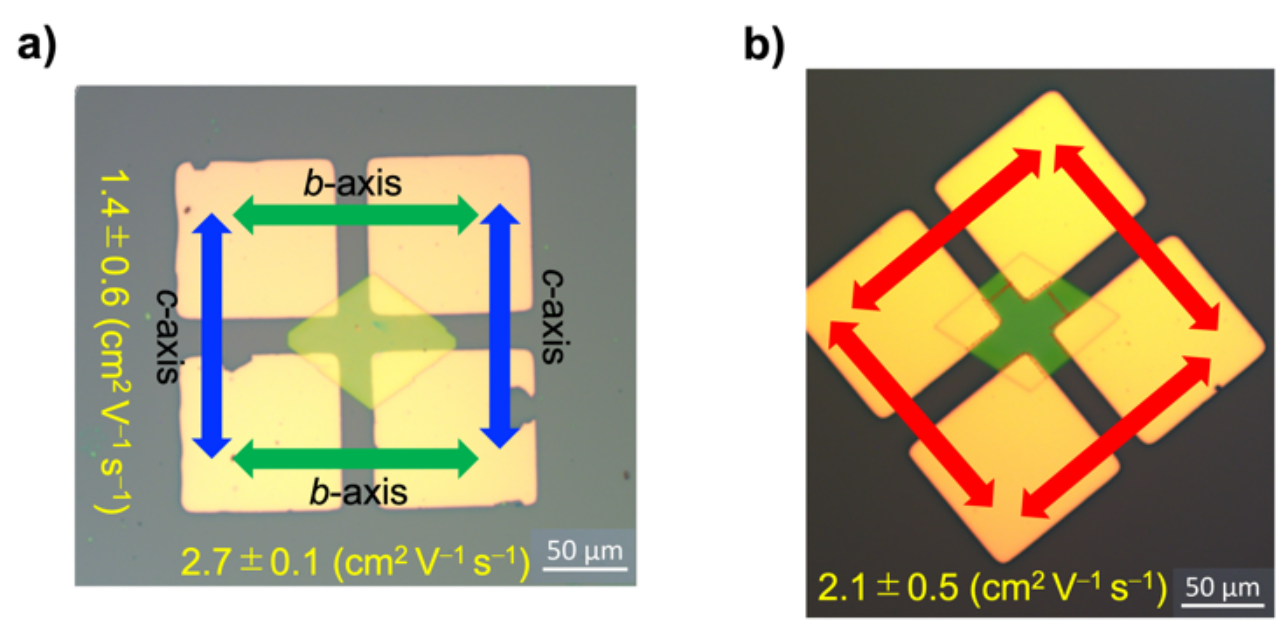

Figure S25. Anisotropic SC-OFET hole mobilities of **Ph-BP** crystals measured a) along the *b*- and *c*-axes and b) along the direction tilted 45° from the *b*-axis.

Table S7. SC-OFET characteristics of Ph-BP crystals measured along the *b*- and *c*-axes and along the direction tilted 45° from the *b*-axis.

Measurement direction	μ_h ($cm^2 V^{-1} s^{-1}$)	V_{th} [a] (V)	I_{on} (A)	I_{off} (A)	I_{on} / I_{off}
b-axis	2.6	–1.7	-1.09×10^{-4}	-4.45×10^{-7}	2.0×10^{2}
	2.8	3.8	-8.04×10^{-5}	-1.09×10^{-6}	74
c-axis	1.0	1.0	-7.21×10^{-5}	-6.34×10^{-7}	1.1×10^{2}
	1.8	5.6	-7.65×10^{-5}	-7.24×10^{-7}	1.1×10^{2}
45° from the *b*-axis	1.9	N. D.	-2.10×10^{-4}	-1.56×10^{-5}	13
	1.5	N. D.	-5.20×10^{-4}	-1.60×10^{-4}	3.2
	1.7	N. D.	-2.90×10^{-4}	-2.46×10^{-5}	12
	1.8	N. D.	-3.10×10^{-4}	-3.00×10^{-5}	10
	2.8	N. D.	-2.80×10^{-4}	-3.19×10^{-5}	8.8
	2.3	N. D.	-2.30×10^{-4}	-3.04×10^{-5}	7.7
	2.8	N. D.	-2.20×10^{-4}	-2.59×10^{-5}	8.5
	2.3	N. D.	-2.00×10^{-4}	-2.36×10^{-5}	8.3

[a] V_{th} could not be reliably determined for the device oriented at 45° from the *b*-axis because of the high off-current.

4. NMR spectra

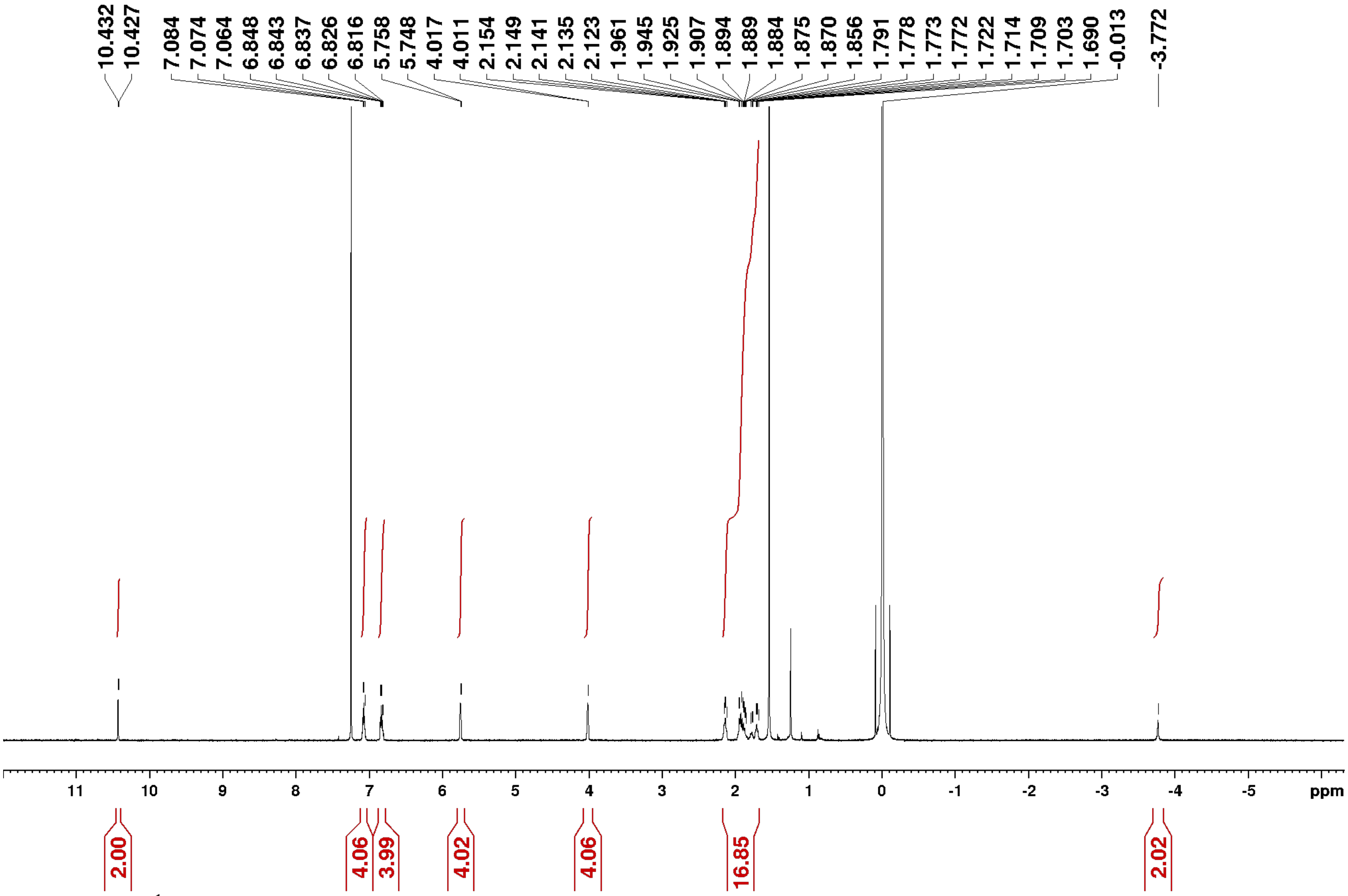

Figure S26. ^{1}H NMR spectrum of **C_6F_5-CP** (600 MHz, $CDCl_3$).

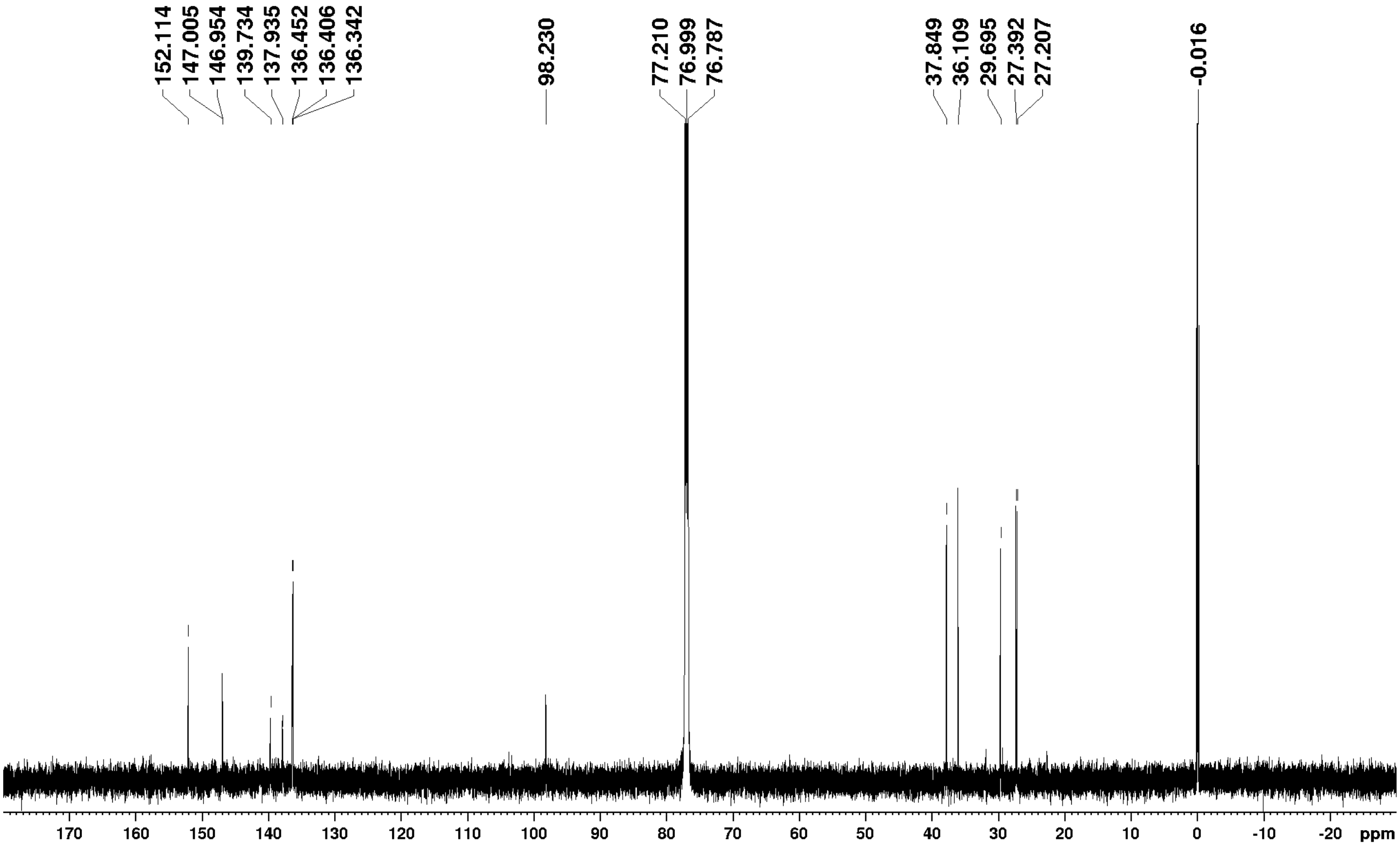

Figure S27. ^{13}C NMR spectrum of **C_6F_5-CP** (151 MHz, $CDCl_3$).

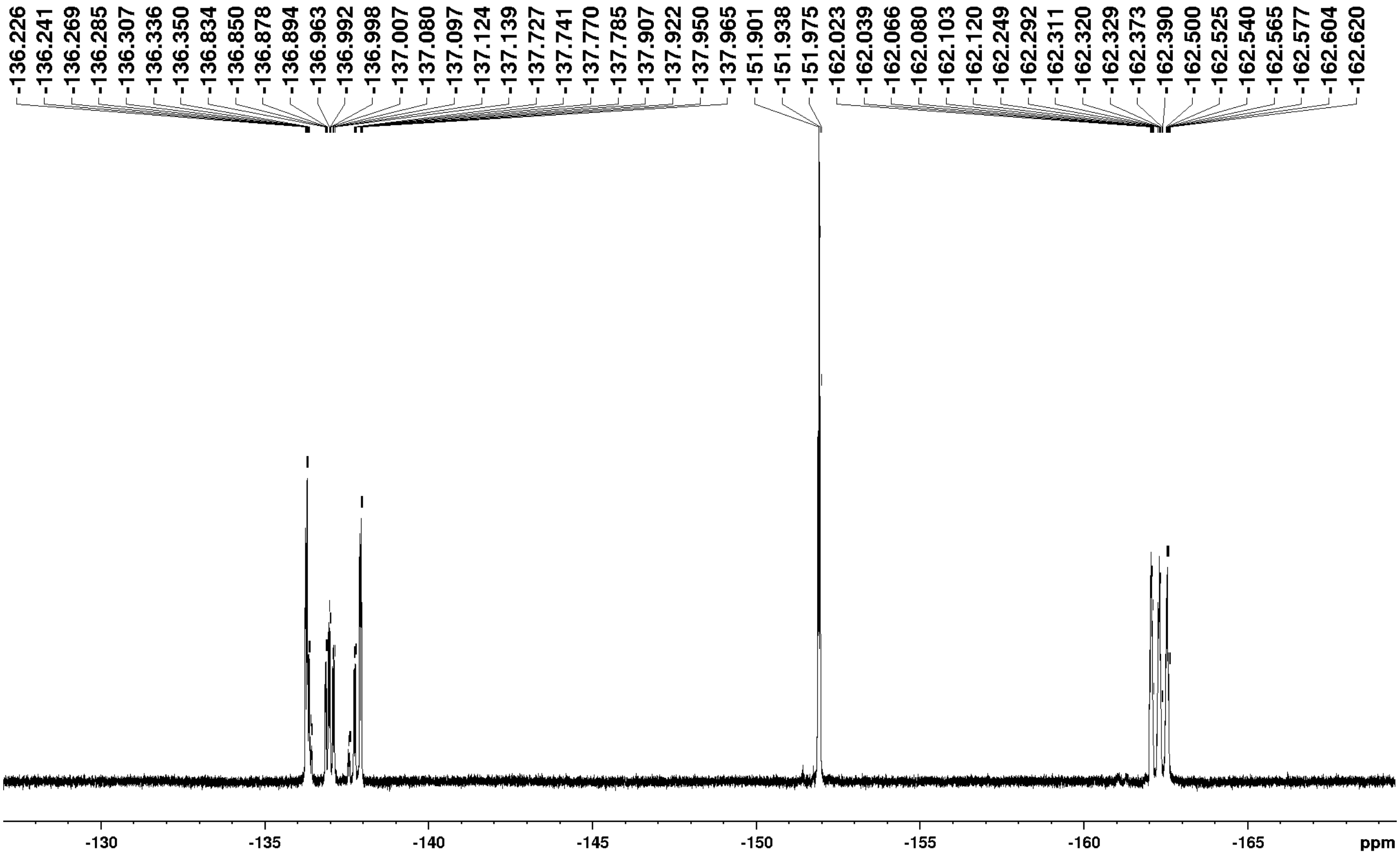

Figure S28. ^{19}F NMR spectrum of **C_6F_5-CP** (565 MHz, $CDCl_3$).

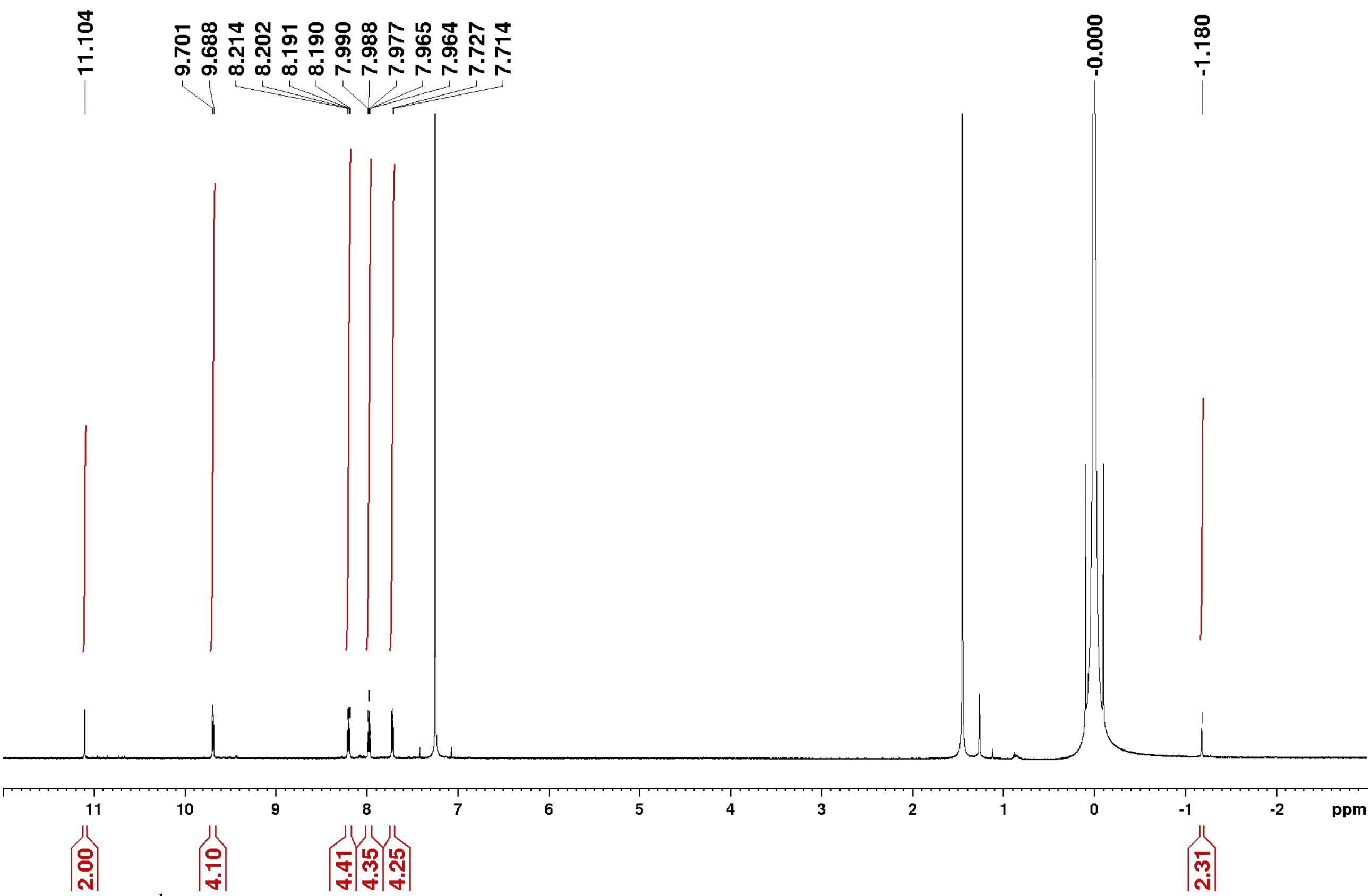

Figure S29. ^{1}H NMR spectrum of **C_6F_5-BP** (600 MHz, $CDCl_3$).

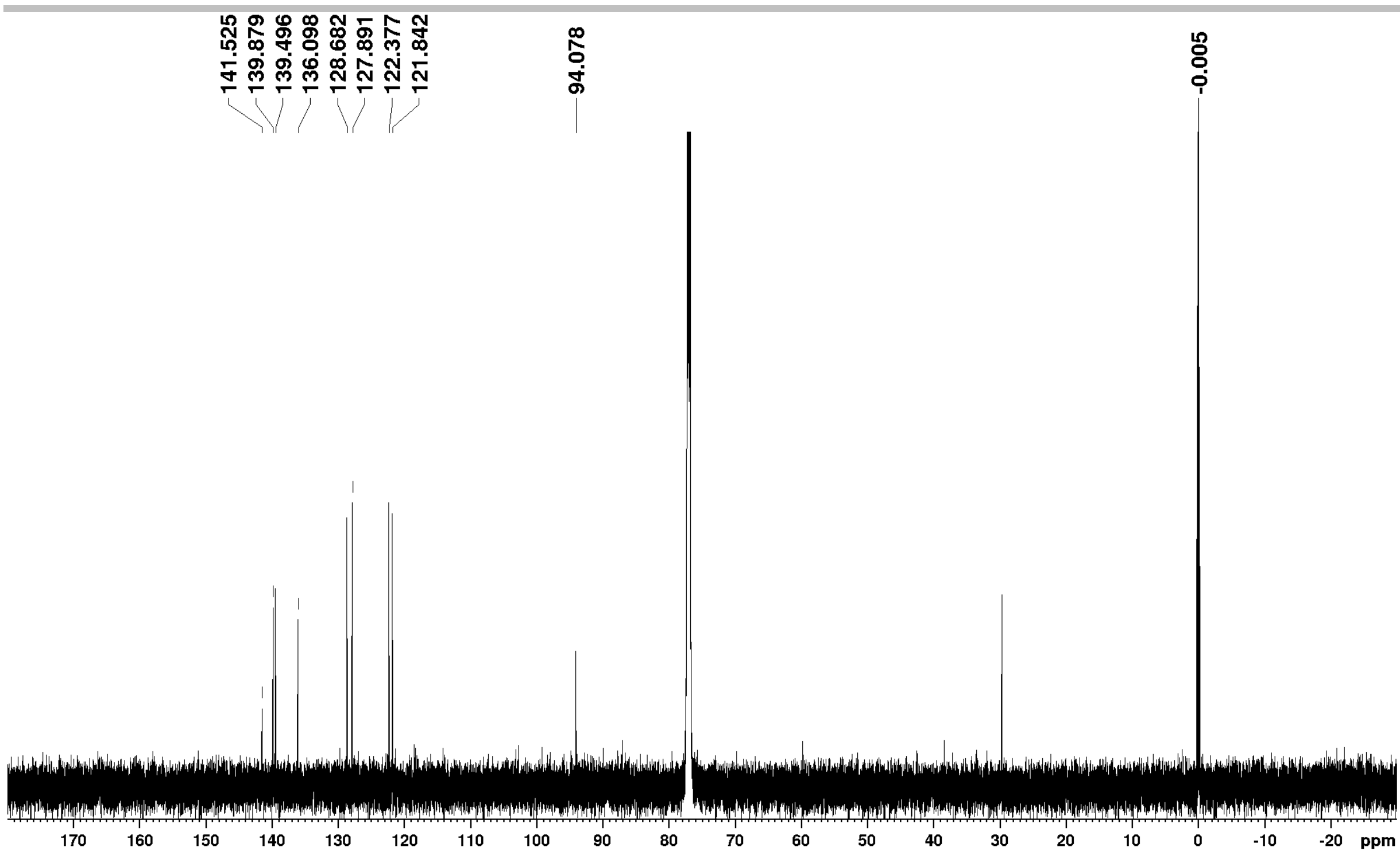

Figure S30. ^{13}C NMR spectrum of **C_6F_5-BP** (151 MHz, $CDCl_3$).

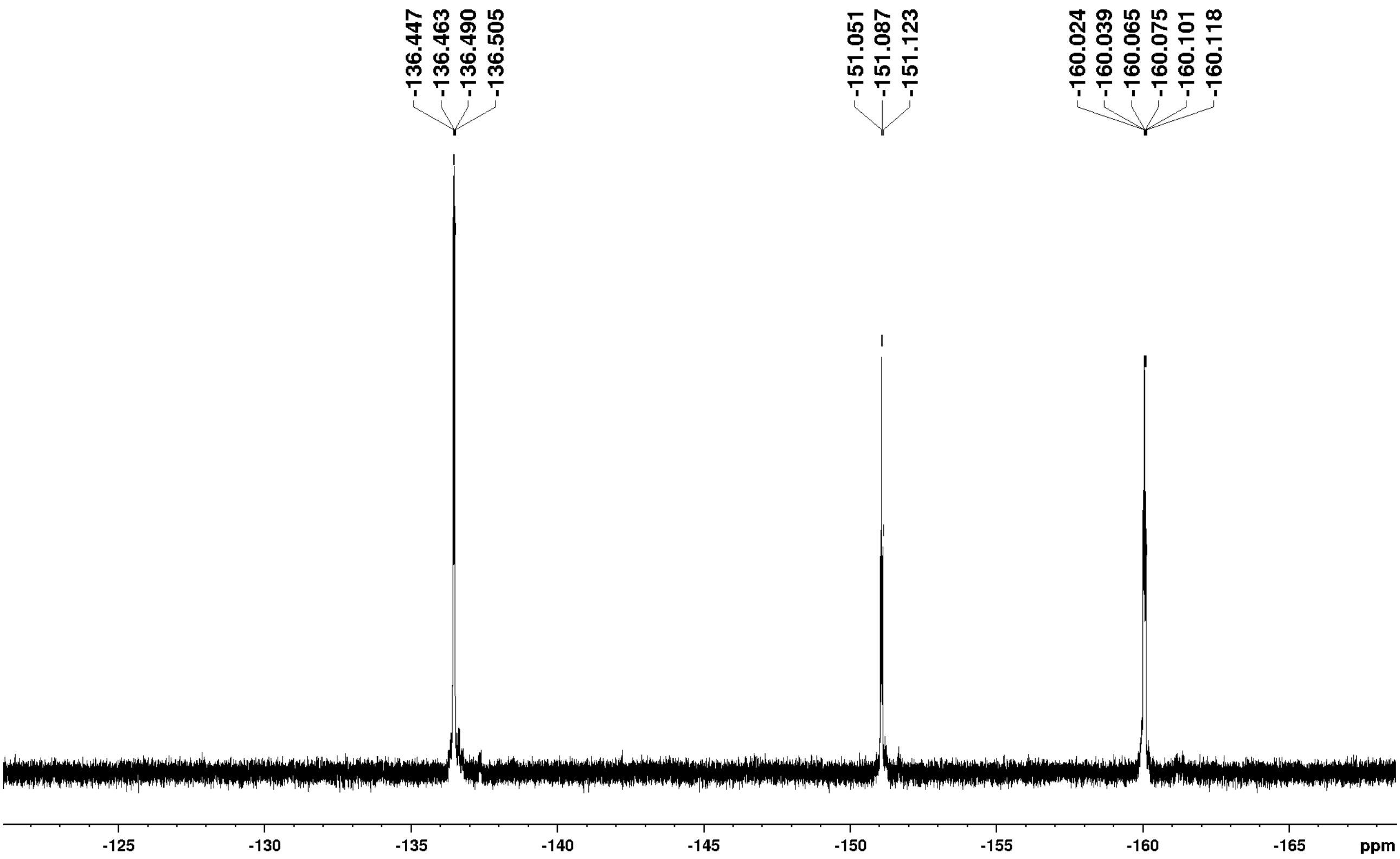

Figure S31. ^{19}F NMR spectrum of **C_6F_5-BP** (565 MHz, $CDCl_3$).

5. HR-MS data

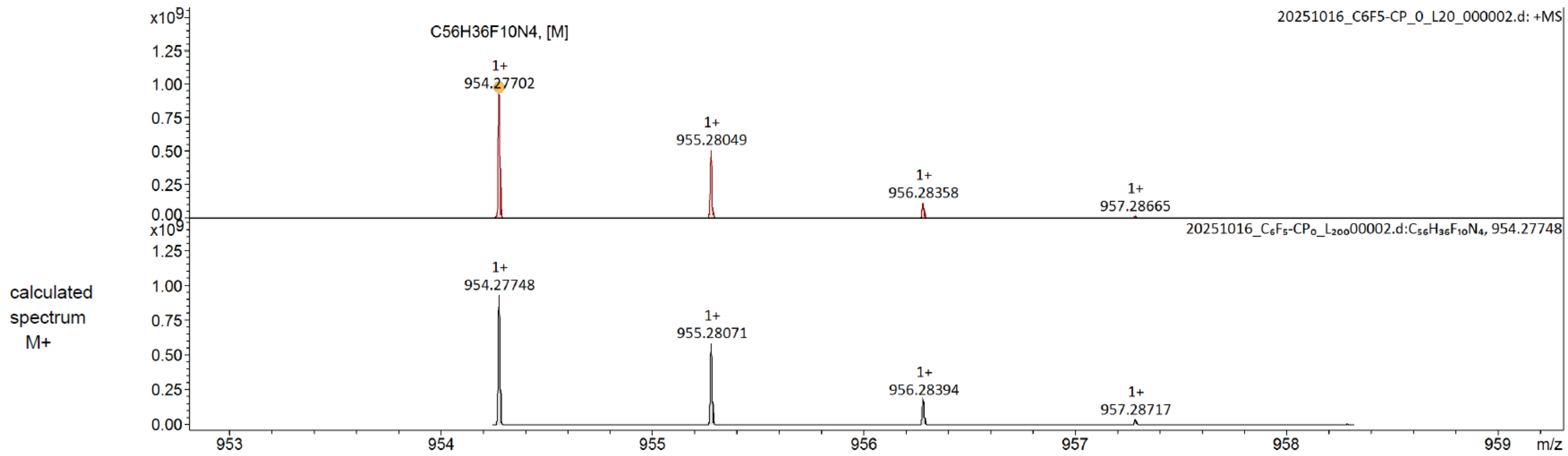

Figure S32. HR-MALDI-TOF MS spectrum of **C_6F_5-CP**.

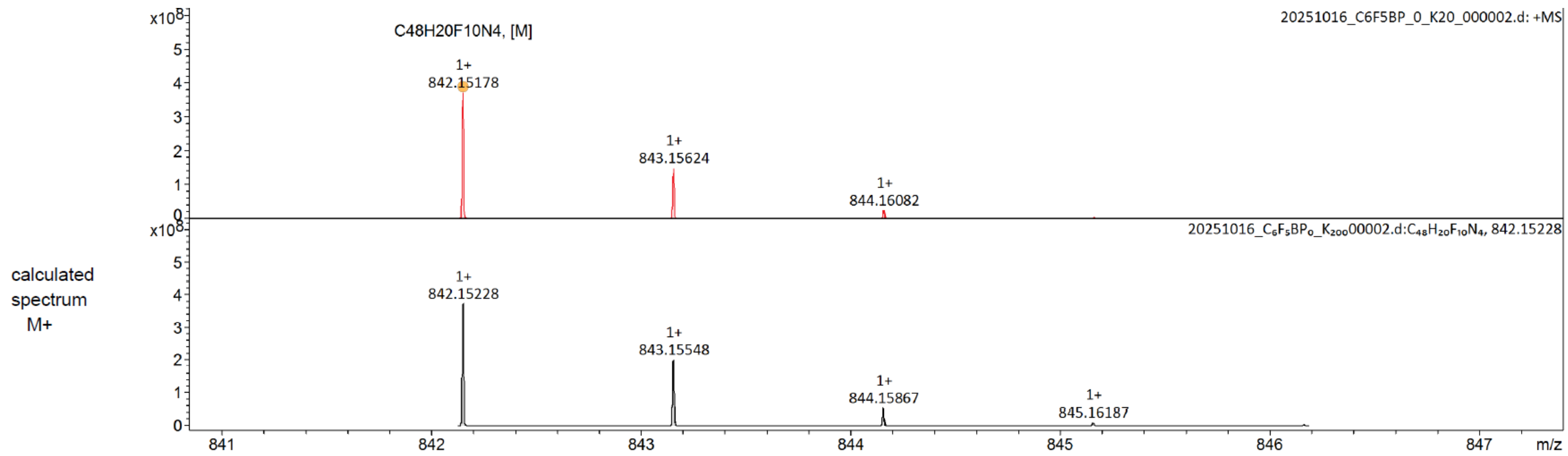

Figure S33. HR-MALDI-TOF MS spectrum of **C_6F_5-BP**.

6. Optimized coordinates

The ESP plots were calculated based on the geometries shown below.

Ph-BP:

C	3.5820312	–2.5715314	–0.0000490
C	2.5089643	–3.4865632	–0.0000484
C	1.2858481	–2.6847246	–0.0000418
N	1.5876133	–1.3614795	–0.0000171
C	2.9504272	–1.2380202	–0.0000240
C	2.4769120	3.4920671	0.0000930
C	3.5618835	2.5793675	0.0000571
C	2.9881043	1.2429581	0.0000280
N	1.6197544	1.4104589	0.0000537
C	1.2490973	2.7287719	0.0000864
C	3.6222869	–0.0011251	–0.0000064
C	–3.5820301	2.5715232	–0.0000493
C	–2.5089656	3.4865570	–0.0000063
C	–1.2858476	2.6847208	0.0000524
N	–1.5876096	1.3614746	0.0000462
C	–2.9504226	1.2380151	–0.0000085
C	–0.0225381	3.2726110	0.0000908
C	–2.4769122	–3.4920702	0.0000259
C	–3.5618838	–2.5793709	0.0000519
C	–2.9881047	–1.2429617	–0.0000033
N	–1.6197539	–1.4104623	–0.0000302
C	–1.2490972	–2.7287748	–0.0000164
C	0.0225380	–3.2726144	–0.0000362
C	–3.6222844	0.0011220	–0.0000143
C	–4.8970364	3.0543422	–0.0001449
C	–5.1061426	4.4303122	–0.0001790
C	–4.0330754	5.3313998	–0.0001217
C	–2.7241809	4.8638729	–0.0000394
C	2.6908106	4.8748090	0.0001230
C	3.9930442	5.3434375	0.0001137
C	5.0756971	4.4449215	0.0000732
C	4.8778300	3.0735334	0.0000455
C	–2.6908111	–4.8748119	0.0000641
C	–3.9930447	–5.3434404	0.0001358
C	–5.0756978	–4.4449245	0.0001761
C	–4.8778304	–3.0735365	0.0001378
C	4.8970358	–3.0543559	–0.0000548
C	5.1061373	–4.4303268	–0.0000611
C	4.0330677	–5.3314110	–0.0000625
C	2.7241749	–4.8638797	–0.0000548
C	–5.1148140	–0.0006586	–0.0000193
C	5.1148164	0.0006644	–0.0000225
C	–5.8209430	–0.0041265	1.2048473

C	–7.2136112	–0.0097681	1.2062632
C	–7.9129773	–0.0126445	–0.0000293
C	–7.2136027	–0.0097599	–1.2063169
C	–5.8209346	–0.0041243	–1.2048910
C	5.8209539	0.0041740	1.2048392
C	7.2136219	0.0098223	1.2062451
C	7.9129793	0.0126715	–0.0000526
C	7.2135962	0.0097513	–1.2063350
C	5.8209280	0.0041030	–1.2048992
H	1.0046623	0.5982535	0.0000313
H	–0.0295896	4.3556112	0.0001088
H	–1.0046620	–0.5982568	–0.0000383
H	0.0295892	–4.3556145	–0.0000369
H	–5.7479452	2.3923832	–0.0002002
H	–6.1212631	4.8103974	–0.0002544
H	–4.2267146	6.3977817	–0.0001480
H	–1.8952071	5.5623765	–0.0000044
H	1.8577407	5.5674694	0.0001536
H	4.1830787	6.4102556	0.0001373
H	6.0875787	4.8329065	0.0000623
H	5.7288445	2.4117870	0.0000120
H	–1.8577412	–5.5674724	0.0000443
H	–4.1830793	–6.4102585	0.0001688
H	–6.0875793	–4.8329096	0.0002406
H	–5.7288449	–2.4117897	0.0001723
H	5.7479475	–2.3924015	–0.0000517
H	6.1212566	–4.8104152	–0.0000642
H	4.2267032	–6.3977936	–0.0000702
H	1.8951990	–5.5623808	–0.0000595
H	–5.2721478	–0.0006487	2.1390874
H	–7.7523100	–0.0113119	2.1467248
H	–8.9967734	–0.0166187	–0.0000332
H	–7.7522948	–0.0112972	–2.1467823
H	–5.2721327	–0.0006491	–2.1391272
H	5.2721653	0.0007222	2.1390832
H	7.7523274	0.0113943	2.1467029
H	8.9967755	0.0166532	–0.0000643
H	7.7522815	0.0112688	–2.1468044
H	5.2721196	0.0005950	–2.1391314

C_6F_5-BP:

C	3.5833070	–2.5691015	0.0000714
C	2.5128094	–3.4866777	0.0000611
C	1.2877749	–2.6876284	0.0000233
N	1.5857781	–1.3630938	0.0000094
C	2.9457366	–1.2384738	0.0000293
C	2.4730977	3.4973185	0.0000552

C	3.5572037	2.5840892	–0.0000242
C	2.9805543	1.2493685	–0.0000020
N	1.6149122	1.4154641	0.0000560
C	1.2451992	2.7344582	0.0000966
C	3.6073142	0.0027521	0.0000003
C	–3.5833070	2.5691014	–0.0000216
C	–2.5128093	3.4866776	0.0000489
C	–1.2877748	2.6876284	0.0000892
N	–1.5857784	1.3630935	0.0000570
C	–2.9457366	1.2384738	0.0000022
C	–0.0261403	3.2774947	0.0001325
C	–2.4730975	–3.4973189	0.0000227
C	–3.5572036	–2.5840899	0.0000340
C	–2.9805547	–1.2493688	0.0000103
N	–1.6149122	–1.4154643	–0.0000040
C	–1.2451990	–2.7344581	0.0000048
C	0.0261403	–3.2774949	0.0000081
C	–3.6073145	–0.0027526	–0.0000059
C	–4.8977196	3.0513887	–0.0001209
C	–5.1111536	4.4266292	–0.0001260
C	–4.0400803	5.3287694	–0.0000386
C	–2.7305153	4.8629351	0.0000446
C	2.6872774	4.8795130	0.0000599
C	3.9893536	5.3484597	–0.0000249
C	5.0716525	4.4505495	–0.0001245
C	4.8718276	3.0796350	–0.0001278
C	–2.6872767	–4.8795134	0.0000413
C	–3.9893529	–5.3484604	0.0000783
C	–5.0716520	–4.4505506	0.0001020
C	–4.8718274	–3.0796359	0.0000812
C	4.8977196	–3.0513885	0.0001433
C	5.1111538	–4.4266290	0.0001870
C	4.0400807	–5.3287693	0.0001632
C	2.7305155	–4.8629351	0.0001038
C	–5.0960721	–0.0039179	–0.0000213
C	5.0960718	0.0039176	–0.0000279
C	–5.8186729	–0.0048475	1.1898447
C	–7.2076102	–0.0037875	1.2042196
C	–7.9027137	–0.0029466	–0.0000482
C	–7.2075872	–0.0039551	–1.2043026
C	–5.8186501	–0.0050142	–1.1899007
C	5.8186841	0.0050335	1.1898311
C	7.2076213	0.0039752	1.2041933
C	7.9027135	0.0029475	–0.0000811
C	7.2075756	0.0037686	–1.2043289
C	5.8186385	0.0048281	–1.1899143
H	0.9987581	0.6042361	0.0000642

H	–0.0343767	4.3603679	0.0001724
H	–0.9987584	–0.6042361	0.0000082
H	0.0343767	–4.3603681	0.0000099
H	–5.7527518	2.3944172	–0.0002018
H	–6.1268338	4.8044163	–0.0002030
H	–4.2356209	6.3944990	–0.0000417
H	–1.9030670	5.5629720	0.0001016
H	1.8545888	5.5723919	0.0001259
H	4.1796498	6.4149459	–0.0000209
H	6.0834272	4.8380990	–0.0002044
H	5.7276484	2.4238936	–0.0002179
H	–1.8545880	–5.5723922	0.0000288
H	–4.1796488	–6.4149467	0.0000927
H	–6.0834265	–4.8381002	0.0001394
H	–5.7276483	–2.4238947	0.0001069
H	5.7527518	–2.3944168	0.0001738
H	6.1268340	–4.8044159	0.0002437
H	4.2356214	–6.3944989	0.0001962
H	1.9030673	–5.5629721	0.0000940
F	5.1740730	0.0069040	2.3616020
F	7.8799464	0.0042281	2.3583811
F	9.2362026	0.0020427	–0.0001061
F	7.8798564	0.0038227	–2.3585425
F	5.1739835	0.0064978	–2.3616610
F	–5.1740512	–0.0065367	2.3616096
F	–7.8799243	–0.0038602	2.3584137
F	–9.2362028	–0.0020412	–0.0000611
F	–7.8798790	–0.0041888	–2.3585098
F	–5.1740057	–0.0068659	–2.3616534

7. References

[1] H. Yamada, K. Kushibe, T. Okujima, H. Uno, N. Ono, *Chem. Commun*. **2006**, 383.
[2] K. Miyazaki, K. Teranishi, H. Matsuda, K. Matsuo, M. Yamauchi, Y. Mizuhata, N. Shioya, T. Hasegawa, H. Yamada, *Adv. Mater. Interfaces* **2025**, *12*, 2400946.
